# Tau oligomers mediate aggregation of RNA‐binding proteins Musashi1 and Musashi2 inducing Lamin alteration

**DOI:** 10.1111/acel.13035

**Published:** 2019-09-18

**Authors:** Mauro Montalbano, Salome McAllen, Urmi Sengupta, Nicha Puangmalai, Nemil Bhatt, Anna Ellsworth, Rakez Kayed

**Affiliations:** ^1^ Mitchell Center for Neurodegenerative Diseases University of Texas Medical Branch Galveston TX USA; ^2^ Department of Neurology, Neuroscience and Cell Biology University of Texas Medical Branch Galveston TX USA

**Keywords:** Musashi, neurodegeneration, nuclear dysfunction, protein aggregation, tau

## Abstract

The exact mechanisms leading to neurodegeneration in Alzheimer's disease (AD) and other tauopathies are not yet entirely understood. However, it is known that several RNA‐binding proteins (RBPs) form toxic aggregates and also interact with tau in such granules in tauopathies, including AD. The Musashi (MSI) family of RBPs, consisting of two homologues: Musashi1 and Musashi2, have not been extensively investigated in neurodegenerative diseases. Here, using a tau inducible HEK (iHEK) model we investigate whether MSI proteins contribute to the aggregation of toxic tau oligomers (TauO). Wild‐type and mutant P301L tau iHEK cells are used to study the effect of different tau variants on the cellular localization of MSI proteins. Interestingly, we observe that tau co‐localizes with MSI in the cytoplasm and nuclei, altering the nuclear transport of MSI. Furthermore, incremental changes in the size and density of nuclear MSI/tau foci are observed. We also report here that TauO interact with MSI to cause the formation of distinct nuclear aggregates. Moreover, tau/MSI aggregates induce structural changes to LaminB1, leading to nuclear instability. These results illustrate a possible mechanism of neurodegeneration mediated by the aggregation of MSI proteins and TauO, suggesting that MSI plays a critical role in cellular dysfunction.

**Abbreviations**
AD: Alzheimer's disease
ALS: amyotrophic lateral sclerosis
C: cytoplasm
CNS: central nervous system
FTD: frontal temporal dementia
HMW: high molecular weight
iHEK: inducible human embryonic kidney cells
IP: immunoprecipitation
IPZ: Importazole
KH: hnRNP K homology domain
LCDs: low‐complexity domains
MSI: Musashi
MSI1: Musashi1
MSI2: Musashi2
N: nucleus
NSC: neuronal stem cells
PCC: Pearson correlation coefficient
RBDs: RNA‐binding domains
RBPs: RNA‐binding proteins
RNA: ribonucleic acid
RNP: ribonucleoprotein
ROI: region of interest
rpS6: ribosomal protein subunit 6
RRM: RNA recognition motif
SGs: stress granules
TDP‐43: transactive response DNA binding protein‐43
TIA‐1: T‐cell intracytoplasmic antigen
WT: wild‐type

## 1 INTRODUCTION

RNA‐binding proteins (RBPs) are proteins that bind to RNA through globular RNA‐binding domains (RBDs), changing the fate or function of the bound RNAs as a result. Hundreds of RBPs have been discovered over the years, and many new proteins have been found with proteome‐wide studies lacking conventional RBDs (Hentze, Castello, Schwarzl, & Preiss, 2018). The RBPs bind to RNA through a limited set of structurally well‐defined RBDs (Lunde, Moore, & Varani, 2007), such as the RNA recognition motif (RRM; Clery, Blatter, & Allain, 2008), hnRNP K homology domain (KH; Nicastro, Taylor, & Ramos, 2015), or DEAD box helicase domain (Linder & Jankowsky, 2011). RBPs also participate in the formation of ribonucleoprotein complexes (RNP) that are mainly involved in gene expression and determining large RNP machines, such as ribosomes (Ramakrishnan, 2014) and spliceosomes (Papasaikas & Valcarcel, 2016).

Eukaryotic cells contain cytoplasmic assemblies of RNAs and RBPs, termed RNA granules. Under stress, such granules start to accumulate in the cytoplasm, which are known as stress granules (SGs). The maturation and accumulation of SGs are a potential source of soluble protein aggregates (Dobra, Pankivskyi, Samsonova, Pastre, & Hamon, 2018). Several RBPs that interact with tau to form toxic granules and aggregates in both Alzheimer's disease (AD) and other tauopathies have recently been identified. For example, transactive response DNA binding protein (TDP‐43), fused in sarcoma, T‐cell intracytoplasmic antigen (Apicco et al., 2018; Vanderweyde et al., 2016), and U1‐70K (Bishof et al., 2018) are known to be dysregulated in neurodegenerative diseases (Maziuk, Ballance, & Wolozin, 2017). Furthermore, for some RBPs, aggregation is mediated by low‐complexity domains (LCDs), such as the prion‐like domain, and it appears to be critical for their involvement in disease progression (Harrison & Shorter, 2017; Kato et al., 2012). RBPs contain intrinsically disordered LCDs that enclose a repeated sequence that is enriched with glycine and uncharged polar amino acids (Banani, Lee, Hyman, & Rosen, 2017). These properties cooperate such that self‐assembly is induced, promoting regulated aggregation of RBPs and client mRNA transcripts to form RNA granules (Anderson & Kedersha, 2008).

In this study, we focused our attention specifically to the Musashi (MSI) RBP family. It contains two homologues: MSI1 and MSI2. MSI proteins constitute an evolutionarily conserved family of RBPs that are preferentially expressed in the stem cells of the central nervous system (CNS; Good et al., 1998). MSI1 plays an important role in regulating asymmetric cell division of neuronal precursor cells through the translational regulation of target mRNAs. The mammalian MSI1 is strongly expressed in fetal and adult neuronal stem cells (Okano et al., 2005). MSI proteins possess two RRMs sharing almost 90% amino acid sequence homology (Nakamura, Okano, Blendy, & Montell, 1994; Okano et al., 2005). The MSI2 pattern of expression in the CNS is comparable to MSI1 in terms of the high expression levels in neural stem/progenitor cells, and they have been postulated to play mutually overlapping roles. Nevertheless, MSI2 differs from MSI1 in that it is expressed continuously in only some of the neurons in the CNS. Also, it has been suggested that MSI2 may play a unique role for generating and/or maintaining specific neuronal lineages (Sakakibara, Nakamura, Satoh, & Okano, 2001).

With the exception of MSIs interaction with tau (Sengupta et al., 2018), as well as its expression in both AD and Pick's disease (Lovell & Markesbery, 2005), no available data have been reported thus far on their roles in neurodegenerative diseases. It has been shown that mutations in RBPs cause amyotrophic lateral sclerosis, frontotemporal dementia, spinocerebellar ataxia, and myopathies. Additionally, some of these disease‐linked RBPs have also been shown to form pathological aggregates (Irwin et al., 2015). Thus, dysfunction of RBPs evidently influences the development of neurodegenerative diseases to a significant degree. Recent results further suggest that the aggregation of RBPs is also a pathological feature in tauopathies. Various RBPs co‐localize with hyper‐phosphorylated tau in patient tissues and progressively accumulate with aggregated tau in mouse models (Maziuk et al., 2018). However, it is unknown as to how and when SGs or RBPs contribute to tauopathies.

We previously reported that the cellular localization of MSI1 and MSI2 in human AD cortical tissues is found in the cytoplasm and nuclei of mature neurons (Sengupta et al., 2018). In this scenario, we demonstrated that tau oligomers (TauO) interact with the MSI proteins in AD brains, and that MSI proteins are able to form oligomers in vitro (Sengupta et al., 2018). Clarifying their cell compartment location and regulation is an important step to identifying MSI’s crucial roles in the pathogenesis of AD.

AD is the most common neurodegenerative disorder associated with structural and functional alterations of neurons, causing progressive decline of memory and other cognitive functions. The aggregation of tau is a defining pathological hallmark of AD (Clavaguera et al., 2013). Tau is a cytosolic protein, distributed in the somatodendritic compartment, but predominantly in the axons (Binder, Frankfurter, & Rebhun, 1985; Papasozomenos & Binder, 1987). It regulates microtubule assembly, stability and axonal transport of vesicles and organelles (Trinczek, Ebneth, Mandelkow, & Mandelkow, 1999). Also, tau has been shown to localize to the ribosomes of both neurons and astrocytes in the AD brain (Papasozomenos, 1989; Papasozomenos & Binder, 1987). This association with ribosomes raised new interesting questions on whether it has any relation to protein synthesis, but this type of function is still not well understood.

It is well established that the toxic tau species involved in neuronal dysfunction and death are the oligomeric forms (Castillo‐Carranza et al., 2014; Gerson et al., 2016; Lasagna‐Reeves, Castillo‐Carranza, Sengupta, Guerrero‐Munoz, et al., 2012; Lasagna‐Reeves, Castillo‐Carranza, Sengupta, Sarmiento, et al., 2012). Different studies have shown many other “noncanonical” locations of tau within the cells. For example, it has been found that nonphosphorylated tau localizes in the cell membrane of different cell lines (Arrasate, Perez, & Avila, 2000; Buee, Bussiere, Buee‐Scherrer, Delacourte, & Hof, 2000). Tau has also been found in the lipid rafts in AD brains (Nishikawa et al., 2016), mouse brains (Kawarabayashi et al., 2004), and primary neurons (Collin et al., 2014). Additionally, it is also present in synapses (DeVos et al., 2018). Recently, pathogenic phosphorylated tau has been discovered to disrupt nuclear‐cytoplasmic transport in AD through the binding of the nuclear pore protein, Nup98 (Eftekharzadeh et al., 2018; Lester & Parker, 2018). All these findings suggest that tau‐associated proteins could change the localization of tau and its function, triggering neurodegeneration and toxic aggregation in different cell organelles. Tau has been localized within the nucleus of wild‐type (WT) mouse brain neurons (Metuzals, Robitaille, Houghton, Gauthier, & Leblanc, 1988; Sultan et al., 2011) and in the nucleus of both AD and control brains (Brady, Zinkowski, & Binder, 1995). Different studies from 1988 to the present have found tau in the nucleus and nucleolus of neurons, and there is strong evidence to support the idea that post‐translational modifications can modulate its nuclear translocation and function. It has also been observed that nucleolar tau plays a role in the formation of heterochromatin of ribosomal DNA (rDNA; Maina, Bailey, Doherty, & Serpell, 2018), which suggests that tau may play a role in rDNA transcription and/or rRNA processing (Bou Samra et al., 2017). However, we are still far from understanding the complete function of nuclear tau. It is also essential to decipher whether nuclear tau typically interacts with and influences DNA, and how it mediates neurotoxicity in neurodegenerative pathologies (Frost, Bardai, & Feany, 2016; Frost, Hemberg, Lewis, & Feany, 2014).

In this study, we report the novel finding of how in vitro MSI proteins mediate nuclear translocation of tau using an inducible HEK system (iHEK), showing aggregation and changes in cellular localization of WT and mutant P301L tau forms. We investigated the effects of tau on MSI and found a surprising co‐localization with MSI in the cytoplasm and nuclei. This study compares the effects of tau in MSI expression, localization, and function in vitro. To study the effect of WT and mutant (P301L tau form) tau on MSI, we used tetracycline (Tet)‐ iHEK cells that express WT human and P310L mutant tau forms. This is a well‐established model to study tau aggregation, propagation, and cellular mechanisms that involve other proteins (Varghese et al., 2016; Woerman et al., 2016). We showed that tau modulates MSI protein levels and regulates their cellular localization and aggregation. This study suggests that the pathophysiology of tauopathies might be complemented by the interaction of tau with MSI RBPs and coupled by a toxic nuclear accumulation.

## 2 MATERIALS AND METHODS

### 2.1 Cell cultures and treatments

Three different cell lines have been used in this study: HEK‐293, iHEK overexpressing WT tau, and iHEK overexpressing mutated P301L tau (Abisambra et al., 2013; Meier et al., 2016). They were maintained in Dulbecco's modified Eagle Medium (DMEM) medium supplemented with 10% FBS at 37°C with 5% CO_2_. To induce overexpression of WT and mutant tau, the iHEK cells were treated with 1 µg/ml of Tet for 24 hr in FBS‐depleted DMEM (Gibco™ LS11965118, Fisher Scientific). After 24 hr, two washes with medium were performed to remove excess Tet and immediately after the cells were collected. Cells were collected and stained immediately after 24 hr. Detachment of cells was performed with Trypsin (Gibco™ Trypsin‐EDTA, 0.25% Phenol red, LS25200114 Fisher Scientific) for 3 min in an incubator and then centrifuged at 100 *g* for 5 min. Cell pellets were harvested and collected for protein fractioning. To inhibit nuclear importin (Importin‐β), three different concentrations of Importazole (IPZ, 2,4‐diaminoquinazoline, Abcam, ab146155—5 mg) were used with the following final concentrations: 10, 20, and 40 µM for 12 hr after preincubation (24 hr) with Tet.

### 2.2 Primary cortical neurons culture

Primary cortical neuronal cultures were prepared and maintained as described previously (Beaudoin et al., 2012). Briefly, cortical neurons were isolated from embryonic day 16–18 P301L mice (Jackson Laboratory, stock # 000664) using Accutase^®^ solution (A6964‐100Ml Sigma‐Aldrich). Dissociated neurons were plated at a density of 2 × 10^5^ cells/ml in a 24‐well plate containing high glucose DMEM (Corning) supplemented with 2% B27 (A3582801, Gibco), 10,000 units/ml penicillin, 10,000 µg/ml streptomycin, and 25 µg/ml amphotericin B (15290018, Gibco). After 2 hr, plating medium was removed from cells and replenished with Neurobasal™ medium (12348017, Gibco) plus 2% B27, 0.5 mM L‐glutamine (SH30034.01, HyClone), 10,000 units/ml penicillin, 10,000 µg/ml streptomycin, and 25 µg/ml amphotericin B supplement. 50% of medium changes were performed every 3–5 days. Cells on day in vitro 10 were used for all experiments.

### 2.3 RNA isolation and RT–qPCR

Total RNA from iHEK cells was extracted using TRIzol reagent following the established protocol. RNA samples for real‐time analysis were quantified using a NanoDrop Spectrophotometer (NanoDrop Technologies) and qualified by analysis on an RNA Nano chip using the Agilent 2100 Bioanalyzer (Agilent Technologies); only samples with high‐quality total RNA were used (RIN: 7.5–10.0). Synthesis of cDNA was performed with 0.5 µg or 1 µg of total RNA in a 20 µl reaction using the reagents in the TaqMan Reverse Transcription Reagents Kit from Life Technologies (#N8080234). qPCR amplifications (performed in duplicate or triplicate) were done using 1 µl of cDNA in a total volume of 20 µl using the iTaq Universal SYBR Green Supermix (Bio‐Rad #1725125). The final concentration of the primers was 300 nM. Relative RT–qPCR assays were performed with either 18S RNA or another housekeeping gene as a normalizer. Absolute analysis was performed using known amounts of synthetic transcript from the gene of interest. All PCR assays were run in the ABI Prism 7500 Sequence Detection System. A list of primers used is available in the Supplemental Material section.

### 2.4 Immunofluorescence of fixed cells and confocal microscopy

Cells on a 24‐well coverslip were fixed with 0.5 ml of 4% PFA/PBS for 15 min. The cells were washed three times in phosphate‐buffered saline (PBS), for 5‐min intervals. They were then permeabilized in 0.5 ml PBS/0.2% Triton X‐100 in phosphate‐buffered saline with Tween 0.5% (PBST) for 5 min. Blocking was done in 0.5 ml of 5% NGS Serum in PBST for 1 hr. Primary antibody was diluted in 5% NGS/PBST for overnight incubation at 4°C and then washed three times in PBS‐T, for 10‐min intervals. Secondary antibody diluted in 5% NGS/PBST was incubated for 2 hr at room temperature. All the secondary antibodies were purchased from Thermo Fisher Scientific and used at 1:800 dilution for staining. After 2° antibody, cells were incubated in DAPI (nuclei staining) diluted 1:10,000 in PBST (5 mg/ml stock solution) for 5 min after the first wash. They were then washed two times with PBST, and once with PBS, 10 min each, prior to mounting coverslips. Coverslips were mounted on glass microscope slides using 8–10 µl Prolong Gold Antifade mounting media with DAPI (Invitrogen, P36941) per coverslip. Slides were naturally dried in a fume hood (or store at 4°C until ready to dry in fume hood). The primary antibodies used in this study for immunocytochemistry are as follows: MSI1 (Abcam, ab52865 1/250), MSI2 (Abcam, ab76148 1 µg/ml), Tau13 (1/200), and T22 (1/300). After three washes with PBS, cells were probed with mouse and rabbit‐specific fluorescent‐labeled secondary antibodies (1:200, Alexa Fluor 488 and 546, Life Technologies). The single frame images and Z‐stacks for 3D rendering and orthogonal view were collected using a Zeiss LSM880 Confocal Microscope and processed with Zeiss Lite Black Software, and Nikon 63x oil immersion objective. Images for quantification of the area, area ratio, integrated density, number of foci, and double extractions were captured with a Keyence BZ‐800 Microscope and analyzed using BZ‐X Analyzer. A Nikon 100X oil immersion objective was used for acquisition.

### 2.5 Nuclear Musashi/tau aggregates quantification

For the measurements on Musashi foci in the nuclei, 10 nuclei from control and treated groups have been imaged and analyzed. To extract the nuclear Musashi signal, we obtained and isolated from Z‐stacks (0.5 µm step size) the nuclear area as the target. To quantify area, area ratio, density, number, diameter, and fluorescence intensities of MSI1, MSI2, and T22 aggregates, we performed using a double extraction from the target areas, using BZ‐X Analyzer Software. Number of nuclei in each group has been selected based on previous study (Ricci, Manzo, Garcia‐Parajo, Lakadamyali, & Cosma, 2015). The results have been collected and represented in graphs. Graphs and appropriate statistics have been performed using GraphPad Prism 6 Software.

### 2.6 Imaging analysis and 3D reconstruction of immunofluorescence

Images were captured with 2.84mp CCD Peltier Cooled Camera and stored for analysis. For 3D rendering (volumetric rendering), visualization, and segmentation, 10 images per group were processed and segmented with Arivis Vision 4D Software. Analysis on nuclei and foci was performed using BZ‐X Analyzer from Keyence Company. For image analysis, 10 nuclei or regions of interest were selected for each set of experiments. Area, area ratio, integrated density, number, and density of foci were measured after threshold and segmentation of images. Intensity profiles were obtained from 10 cytoplasm and 10 nuclei for each condition. The measurements of all parameters analyzed were corrected for the elongation factor; in particular, to estimate it we used fluorescent nanospheres 4 µm of diameter (TetraSpeck™ Fluorescent Microspheres Sampler Kit #T‐7284, ThermoFisher Scientific) and calibrate the images acquired. Graphs and statistics were obtained and performed with GraphPad 7.0 Software.

### 2.7 Western blotting and cytoplasm/nucleus fractioning

Western blot analyses were performed with iHEK cell samples. Approximately 10 µg of protein preparations were loaded on precast NuPAGE 4%–12% Bis‐Tris gels (Invitrogen) for SDS‐PAGE analyses. Gels were subsequently transferred onto nitrocellulose membrane and blocked overnight at 4°C with 10% nonfat dry milk. Membranes were then probed for 1 hr at room temperature with α‐MSI1 (1:1,000, ab52865 Abcam), α‐MSI2 (1:1,000, ab76148 Abcam), Pan‐Tau (Tau13—1:10,000, MMS‐520R Covance), GAPDH (1:1,000, ab9485 Abcam), LaminB1 (1:1,000, ab133741 Abcam), and Histone3 (1:1,000, ab201456 Abcam) antibodies diluted in 5% nonfat dry milk. α‐MSI1 immunoreactivity and α‐MSI2 immunoreactivity were detected with an HRP‐conjugated anti‐rabbit IgG (1:6,000, GE Healthcare). Tau13 immunoreactivity and GAPDH immunoreactivity were detected using an anti‐mouse IgG (1:6,000, GE Healthcare) diluted in 5% milk. ECL plus (GE Healthcare) was used to visualize the bands. LaminB1 and GAPDH were used to normalize and quantify the amount of nuclear and cytoplasmic proteins, respectively. The compartment extraction was conducted with Qproteome Cell Compartment Kit (Qiagen, #37502), and nuclear, cell membrane, and cytoplasmic proteins were isolated and preserved for Western analysis.

### 2.8 Tau oligomers production, labeling, and cell treatments

The TauO were produced and characterized following established and published protocols (Gerson, Sengupta, & Kayed, 2017). Tau oligomer labeling was conducted as follows: 1 mg of Alexa Fluor™ 568 NHS Ester (Invitrogen, #A20003) was dissolved in 0.1 M sodium bicarbonate to make the final concentration 1 mg/ml. The dye was then incubated with TauO in a 1:2 (w/w) ratio. The mixture was rotated overnight at 4°C on an orbital shaker. The following day, the solution was centrifuged (30 min, 15,000*g*) using 10 kDa Amicon Ultra‐0.5 Centrifugal Filter Units to remove unbound dye. The oligomers were then washed with 1× PBS until the flow through solution was clear. The filter compartment was then flipped and centrifuged to collect the concentrate. The oligomers were reconstituted to their original volume. TauO‐568 was re‐suspended in complete DMEM to obtain 0.5 and 2 µM final concentration solutions. The cells were treated with tauO for 1 hr at a controlled temperature of 37°C and 5% CO_2_. After, the medium was removed and the cells collected for cytoplasm and nuclear protein fractioning and immunofluorescence assays.

### 2.9 Co‐immunoprecipitation

Immunoprecipitation (IP) with MSI1 (SC‐135721) and MSI2 (ab76148) antibodies has been conducted in cytoplasmic and nuclear fraction of P301L tau iHEK. Cytoplasm/Nucleus fractioning has been described in Western blotting section. In particular, PierceTM Co‐Immunoprecipitation Kit (Thermo Scientific, #26149) was used following manufacturer's guidelines. Briefly, amine‐reactive resin was coupled with MSI1 (Santa Cruz Biotechnology, 69‐Q) and MSI2 antibodies suitable for IP followed by incubating with cytoplasm and nuclear extracts. Bound proteins were eluted in 0.1 M glycine (pH 2.8), adjusting the final pH to 7.0 by adding 1 M Tris‐HCl (pH 8). Isolated fractions were subjected to buffer exchange and finally collected in sterile PBS followed by Western characterization. The total protein concentration was measured with bicinchoninic acid protein assay (Micro BCA Kit, Pierce).

### 2.10 MSI1 silencing

2.5 × 10^5^ cells were seeded into six‐well plates and incubated for 24 hr with complete DMEM. After 24 hr, MSI1 Gapmers (QIAGEN, LG00214872‐EDB) transfections were carried out following the Lipofectamine 2000 (Fisher Scientific #11668–027) datasheet preparation freshly using a final Gapmers concentration of 50 nM per well, and after 5 min of incubation at r.t. the cells were incubated for 24 hr with Lipofectamine/Gapmers solution in DMEM medium without FBS. After 24 hr, the untreated and Gapmers transfected cells were collected and total lysate obtained for Western blot analysis of MSI1, MSI2, and Tau13 (Internalization of labeled Gapmers has been observed after 1 hr of treatment). (Table S1)

### 2.11 Statistical analysis

All in vitro experiments were performed in at least three biological replicates. All data are presented as means ± *SD* and were analyzed using GraphPad Prism Software 7.0. Statistical analyses included Student's *t* test or one‐way ANOVA followed by Tukey's multiple comparisons test. Column means were compared using one‐way ANOVA with treatment as the independent variable. Group means were compared using two‐way ANOVA with factors on treatment, respectively. When ANOVA showed a significant difference, pair wise comparisons between group means were examined by Tukey and Dunnett multiple comparison tests.

## 3 RESULTS

### 3.1 Wild‐type and mutant tau translocate into the nuclei

**Figure 1:**
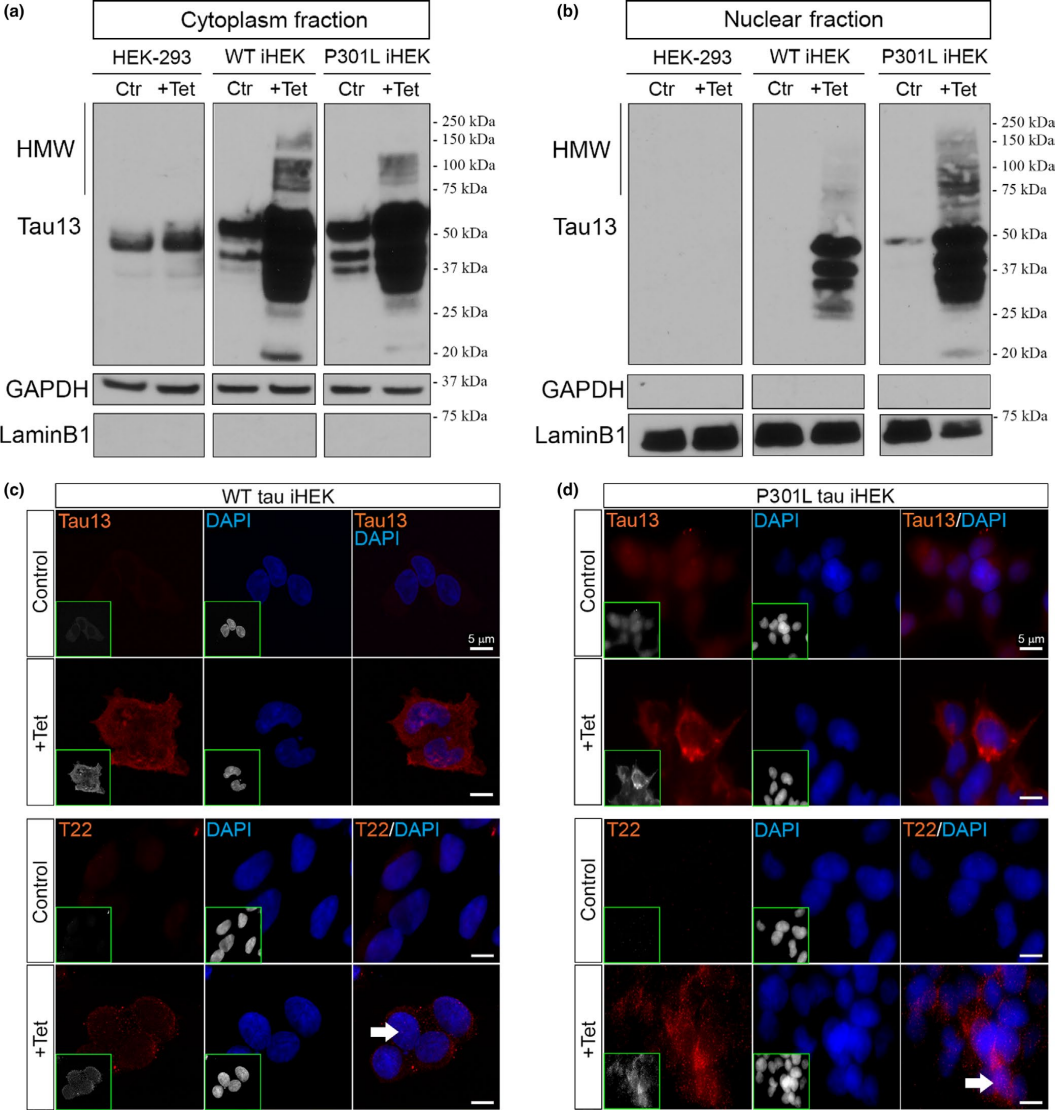
WT and P301L tau overexpression in iHEK cell. Western blot of cytoplasmic and nuclear fractions (control and treated (+Tet) cells). Tau species have been revealed using Tau13 Ab in HEK‐293, WT iHEK, and P301L tau iHEK. (a) We observed an increment (as expected) of tau expression and oligomer occurrence in the cytoplasm fractions of WT and P301L tau iHEK. (b) Nuclear tau species, including oligomers, appear after induction with Tet; in the controls, tau is absent (HEK‐293 and iHEK WT) or at low level (P301L tau iHEK). GAPDH and LaminB1 have been used for the purity of cytoplasmic and nuclear extracts, respectively. (c) Representative confocal images of iHEK WT cells, control and treated, stained with Tau13 (1/200, top panel) and T22 (1/300, bottom panel) antibodies, showing increment of signal in treated cells (100× magnification, zoomed 2×, white scale bar: 5 µm), nuclei have been stained with DAPI (blue). (d) Representative confocal images of iHEK P301L tau cells, control and treated, stained with Tau13 (top panel) and T22 (bottom panel) antibodies, showing increment of signal in treated cells (100× magnification, optical zoom 2×, white scale bar: 5 µm), nuclei has been stained with DAPI (blue). On the bottom left of each fluorescence image, the red and blue channels are shown in gray to better observe tau patterns in the cells

iHEK cells were used to study MSI1 and MSI2 expression and localization after induction with Tet. The levels of tau protein and its gene expression in the iHEK cells carrying WT and mutant (P301L) tau were confirmed by Western blot and RT–qPCR, respectively (Figure S1). Tau forms, in Western blot, were detected using α‐Pan‐tau antibody (Tau13). The Western blot of total cell lysates confirmed the expression of WT (Lane 4) and P301L mutant tau (Lane 6; Figure S1). As expected, in control cells (HEK‐293), tau showed comparable protein levels before and after Tet induction (Lanes 1 and 2). Different tau forms were detected in treated cells by an increments of its oligomeric forms at high molecular weight (HMW, from 75 up to 250 kDa), but not in their respective controls. As a positive control, we used AD brain‐derived tauO (Lasagna‐Reeves, Castillo‐Carranza, Sengupta, Guerrero‐Munoz, et al., 2012) that have been detected by Tau13 (Lane 7; Figure S1). The increment of tau protein, after Tet induction, is due to an increment of MAPT gene expression, measured by RT–qPCR (Figure S1). To study tau cellular localization, we performed a cell fractioning of iHEK models, purifying cytoplasmic and nuclear fractions. Western blotting was used to evaluate cellular compartmentalization of tau using the Tau13 antibody and to identify tau species in these compartments. As expected, HEK‐293 cytosolic fraction did not show difference in tau expression (Lanes 1–2). On the contrary, after induction, in the cytoplasmic fractions of WT and P301L tau cells we observed a marked increase in tau forms (Figure 1a, Lanes 4 and 6). Prior to Tet induction, we did not observe nuclear tau forms in either control or induced HEK‐293 groups (Figure 1b, Lanes 1–2). Surprisingly, after induction, we detected a strong signal of different forms of tau, including HMW, in the nucleoplasm of WT and P301L tau iHEK (Figure 1b, Lanes 4 and 6). These results suggest that tau expression increases nuclear translocation and formation of different tau species, including HMW forms in the nuclei of HEK cells. Specifically, we saw higher level of tau in the P301L tau nuclei then WT nuclei, suggesting that P301L tau forms have high propensity to nuclear translocation. This increment in tau expression has been confirmed and compared by immunofluorescence. Cells were stained with Tau13 and T22 (Lasagna‐Reeves, Castillo‐Carranza, Sengupta, Guerrero‐Munoz, et al., 2012; Lasagna‐Reeves, Castillo‐Carranza, Sengupta, Sarmiento, et al., 2012; Sengupta et al., 2017) an α‐oligomeric tau antibody in WT and P301L tau iHEK fixed cells (Figure 1c,d, respectively). The immunofluorescence staining confirmed the highest signal of tau and its oligomers in induced cells with a predominant localization in the cytoplasm, but with detectable nuclear signals (white arrows). Tau13 staining showed a diffuse signal and T22 detected the oligomeric forms of tau represented with a spotted pattern. α‐GAPDH (cytosol fraction) and α‐LaminB1 (nuclear fraction) were used to verify the purity of the fractions.

### 3.2 High level of tau modulates MSI expression and cellular localization

**Figure 2:**
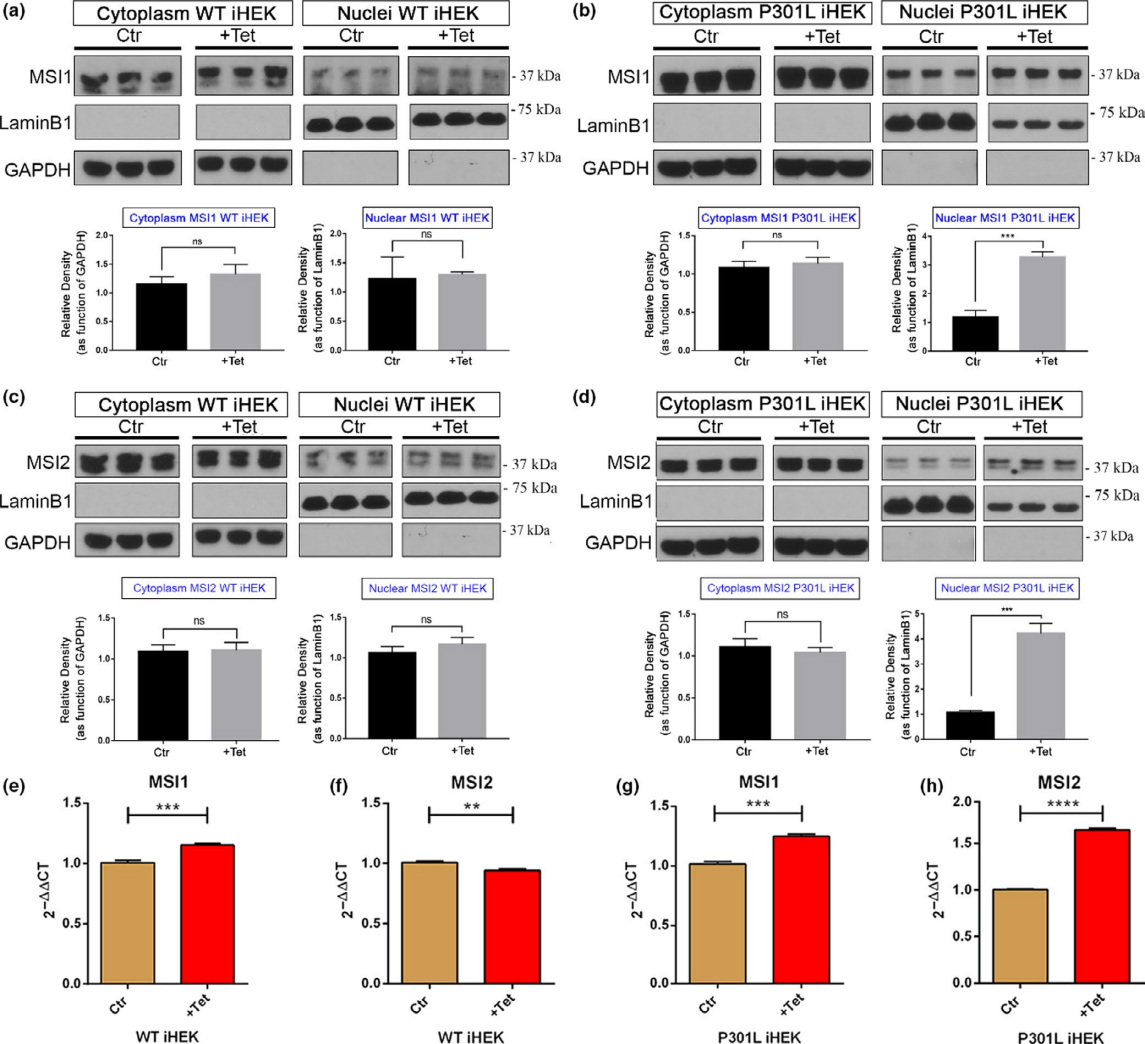
MSI1 and MSI2 in HEK cellular fractions. Western blot of MSI1 in control and treated iHEK WT and iHEK P310L cells. (a) In iHEK WT no significant difference has been observed between cytoplasm (ns, *p = *.2405) and nuclei (ns, *p = *.7448) after treatment with Tet. LaminB1 and GAPDH have been used for purity controls for nuclear and cytoplasm fractions, respectively. (b) Western blot of MSI1 in control and treated P301L tau iHEK. As observed in WT cells, MSI1 did not show significant change in the cytoplasm (ns, *p = *.4573); however, a significant increment has been observed in the nuclei (***p = .0003). (c) Western blot of MSI2 in control and treated iHEK WT tau did not show significant differences in the cytoplasm (ns, *p = *.8293) and in the nuclei (ns, *p = *.1662). (d) In P301L tau iHEK, no significant difference has been observed in the cytoplasm (ns, *p = *.3755) after treatment with Tet for MSI2 but significantly higher in the nuclei (***p = .0002), as observed for MSI1. LaminB1 and GAPDH have been used for purity controls for nuclear and cytoplasm fractions, respectively. (e) RT–qPCR of MSI1 in WT iHEK showed increment in gene expression (***p = .0006). (f) RT–qPCR of MSI2 in WT iHEK showed decrement in gene expression (**p = .0023). (g) RT–qPCR of P301L tau iHEK showed significant increment in MSI1 gene expression (***p = .0001). (h) RT–qPCR in P301L tau iHEK showed significant increment in MSI2 gene expression (****p < .0001). Student's *t* test has been use to determine statistical significance. Error bars represent *SD*

MSI protein localization has been previously investigated and observed in the cytoplasm and nucleoplasm of many cell types, including neurons (Kawahara et al., 2008). Previously, for our knowledge, only Chavali PL. et al. studied MSI1 in HEK293 cells (Chavali et al., 2017). With Western blots of cell fractions, we elucidated MSI’s localization in iHEK cell lines after tau expression. Cytoplasm/Nuclei fractioning has been performed from fresh cell pellets, and with Western blotting of the aforementioned fractions, we evaluated changes in MSI protein levels. We used primary antibodies against MSI1 (MW: 38 kDa) and MSI2 (MW: 35 kDa, present as double band) and with GAPDH (cytosol fraction) and LaminB1 (nuclear fraction) as purity of the fractions. In cytoplasmic and nuclear fractions of WT iHEK, we did not observe a significant difference of MSI1 (Figure 2a) and MSI2 levels (Figure 2c), as showed by respective quantifications. We observed, instead, a significant increment of MSI1 (Figure 2b) and MSI2 (Figure 2d) levels in the P301L tau iHEK nuclear fraction. These observations suggested that mutant tau induces MSI’s nuclear import. We evaluated, with RT–qPCR, if the high tau level could determine some effects on MSI gene expressions. We observed, in WT iHEK, that gene expression of MSI1 increased significantly (Figure 2e), while MSI2 appeared to be down‐regulated (Figure 2f). Interestingly, in P301L tau iHEK, we observed a significant up‐regulation of gene expression of MSI1 and MSI2 (Figure 2g,h, respectively). These results suggest that WT expression and mutant tau expression affect MSI gene expression in different ways, as though the mutant form of tau induces more changes in MSI expression and cellular localization. We evaluated total cell lysates to determine the levels of MSI1 and MSI2 before and after Tet treatment (Figure S2), and we did not observe evident changes in protein levels between the groups. MSI1 levels are higher in P301L than WT iHEK (Figure S2), but no significant differences were observed between controls and treated cells. MSI2 also did not show any significant difference between P301L and WT cells. However, it showed significantly higher in P301L +Tet cells than control cells (Figure S2). These results for MSI1 were confirmed by RT–qPCR in P301L and WT controls, as well as Tet‐treated cells (Figure S2). MSI2 showed an increase in gene expression between controls and Tet‐treated cells (Figure S2). This difference has not been observed at the protein level, suggesting that large amounts of MSI2 transcripts are not translated. We also verified that MSI1 and MSI2 in iHEK‐293 did not change before and after induction with Tet (Figure S2). No significant differences in protein levels in the cytoplasm and nuclear compartments were observed. In addition, a general comparison between control and treated cells confirmed that cytoplasmic MSI1 levels did not change (Figure S2), while only nuclear MSI1 and MSI2 in P301L tau iHEK cells showed a significant increase in their levels (Figure S2).

### 3.3 Tau regulates MSI aggregation and accumulation

**Figure 3:**
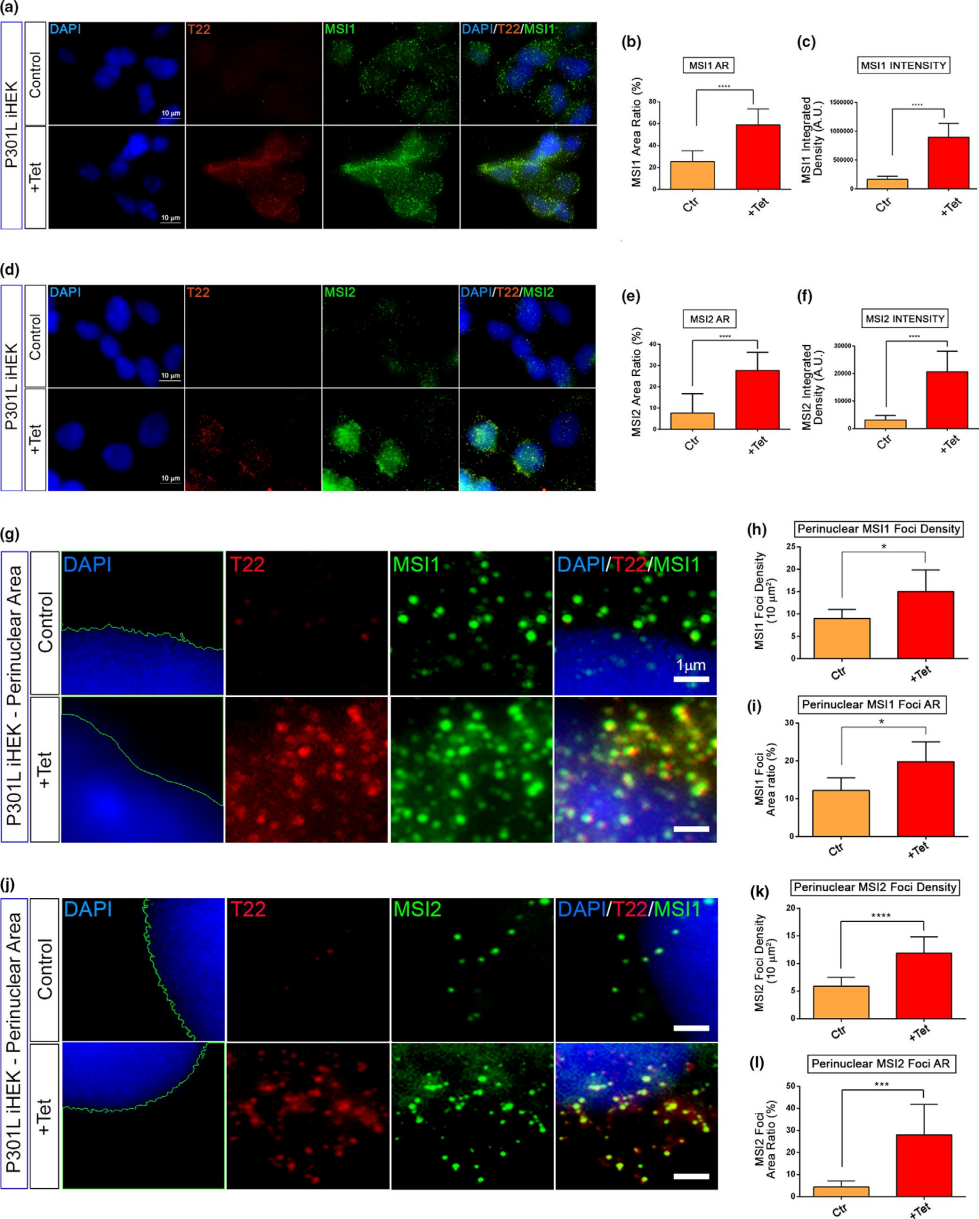
P301l tau increments perinuclear MSI1 and MSI2 foci density. (a) Representative confocal images of P301L tau iHEK control and +Tet stained with MSI1 (green), T22 (red), and nuclei (DAPI—blue). The expected increment of tau oligomers signal has been observed, coupled with a general increment of MSI1 signal (magnification 100×, zoom 2× and white scale bar: 10 µm). (b) MSI1 area ratio (%) has been shown to be significantly increased, (*p < .05) as well as the intensity of MSI1 (c) in the cells (*p < .05). Student's *t* test has been use to determine statistical significance. Error bars represent *SD*. (d) Representative confocal images of P301L tau iHEK control and +Tet stained with MSI2 (green), T22 (red), and nuclei (DAPI in blue). The general increment of MSI2 signal has been observed majorly in the nuclei of the cells (magnification 100×, zoom 2× and white scale bar: 10 µm). (e) Comparing control with treated cells, the area ratio (%) of MSI2 signal has been observed significantly increased (****p < .0001) as well as the intensity of MSI2 (f) inside the cells (****p < .0001). These parameters have been measured and presented in a bar graph. Student's *t* test has been use to determine statistical significance. Error bars represent *SD*. (g) Representative confocal images of P301L tau iHEK perinuclear area of control and +Tet cells stained with MSI1 (green), T22 (red), and nuclei (DAPI—blue). As observed in Figure 4a, expected increment of tau oligomers has been observed as well as a strong increment of MSI1 foci in the perinuclear zone indicating that tau overexpression mediates MSI1 vicinity or import in the nuclei (magnification 100×, zoom 10× and white scale bar: 1 µm). (h) It was observed a significant increment of their density (Ctr vs. +Tet, *p < .05) (i), and area ratio, (Ctr vs. +Tet, *p < .05), these parameters have been measured and presented in a bar graph. (j) Representative confocal images of P301L tau iHEK perinuclear area of control and +Tet cells stained with MSI2 (green), T22 (red), and nuclei (DAPI—blue, delimitated by dashed green line). (k–l) Comparing control with treated cells the density of MSI2 and area ratio (%) significantly higher in the treated cells (****p < .0001 and ***p = .0001, respectively). These parameters have been measured and presented in bar graph (mean ± *SD*). Student's *t* test has been use to determine statistical significance, and error bars represent *SD*. (Magnification 100×, optical zoom 10× and white scale bar: 1 µm)

To evaluate MSI and TauO cellular localization, co‐immunofluorescence of MSI1 and MSI2 with T22 antibody was performed in P301L tau iHEK cells. Interestingly, we observed a significant increase in the signals of both MSI proteins after induction (Figure 3a,d). As expected, we also observed T22 signal in induced cells. We quantified the area ratio (%) and fluorescence intensity to determine these differences. The MSI1 and MSI2 area ratio, as well as their integrated density, were measured by BZ‐X Analyzer Software to confirm the trends observed in the nuclear fraction by Western blot. Immunofluorescence of MSI1 (Figure 3a) showed an increase in signal, represented by a spotted distribution, mainly localizing in the perinuclear and nuclear area. This observation has been confirmed by measuring the area ratio and intensity of MSI1 in cells treated with Tet that showed a significant increase in these measures (Figure 3b‐c). The cells stained with T22, also showed a similar pattern of distribution, in particular, a spotted signal. Similar results have been observed for MSI2 (Figure 3d) with significant increase in area ratio and fluorescence intensity (Figure 3e‐f). The increase in MSI signal was also observed in P301L tau iHEK cells by confocal microscopy, and an orthogonal view clearly revealed abundant nuclear MSI/tauO co‐localization (Figure S3). Furthermore, we also analyzed perinuclear density and area ratio of MSI1 and MSI2 foci in control and treated cells to verify the increase in these proteins’ levels in nuclear proximity. We stained for tauO with T22 antibody and quantified the density of foci for MSI1 and MSI2. We observed an increase in the proximity of nuclei (Figure 3g) and increased foci size density and area ratio for MSI1 (Figure 3h–i). We observed, but with a different magnitude, an increment of nuclear proximity of MSI2 foci (Figure 3j) with significant increase in area density and area ratio (Figure 3k‐l). All these results suggest that P301L tau induces a massive accumulation of MSI foci in the proximity of nuclei with an increment in density. The RT–qPCR results demonstrated an increment of MSI gene expression in P301L tau cells, and we observed an increment of signal and number of MSI foci, which suggests that tau modulates MSI at different levels ranging from expression to aggregation. For these evidences in iHEK cells, we propose a tau‐dependent aggregation effect on MSI proteins.

### 3.4 Tau and MSI form nuclear aggregates

**Figure 4:**
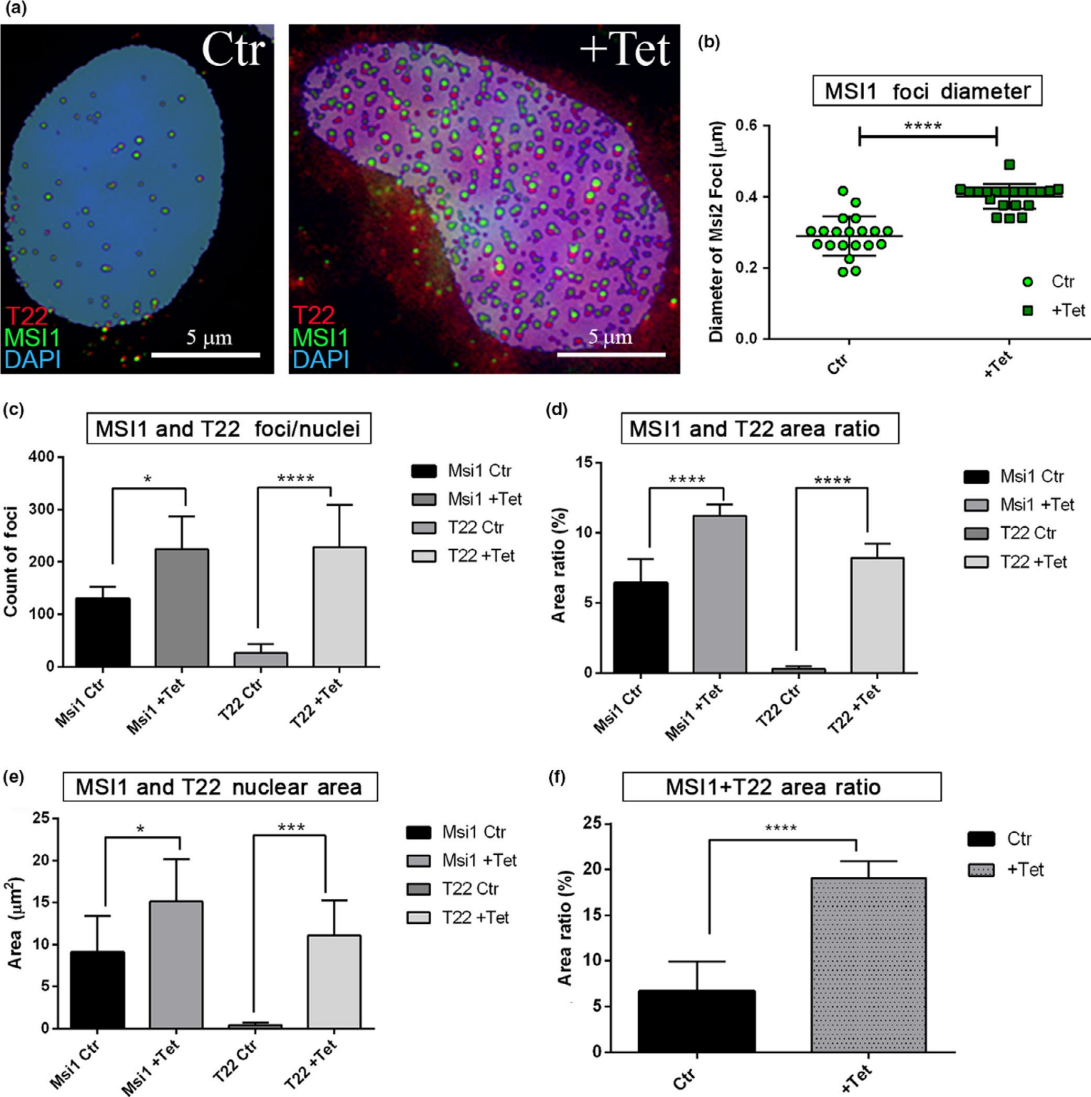
Increment of MSI1 and T22 nuclear foci in P301L tau iHEK. (a) Representative confocal images of control and +Tet nuclei stained with MSI1 (green), T22 (red), and DAPI (blue). Images are obtained after double extraction analysis to quantify their number, area ratio (%), area (µm^2^), and total area ratio. In the confocal images, we can observe the increment of MSI1 and T22 foci in the nucleus after the mutant tau overexpression. Rounded foci look to be the most representative forms observed. (b) The diameter of MSI1 foci has been measured and represented in a dot plot showing a significant increment of the diameter after treatment with Tet (****p < .0001). (c) Increment of number per nuclei of MSI1 (*p = .0440) and T22 (****p < .0001) foci has been observed as well as significant increment of area ratio of MSI1 (****p < .0001) and T22 (****p < .0001) shown in (d). (e) The same trend has been observed in the area covered by MSI (*p = .0368) and T22 (***p = .0003). (f) Increment of coupled signals has been showed with the sum of MSI1 and T22 signal area ratio (from 6.70 ± 3.2% to 19.04 ± 1.86%, ****p < .0001)

To evaluate the presence of MSI1 in the different cell compartments, 3D‐rendering analysis with Arivis Vision 4D Software of MSI1 signal in P301L tau iHEK was performed. The results showed that MSI1 occupied mainly three different cellular compartments: the cytoplasm (Figure S4), nuclear membrane (Figure S4), and nucleoplasm (Figure S4). In the zoomed image, we observed that MSI1 was organized in sub‐rounded structures in the nuclei (Figure S4). We have shown increased amounts of MSI1 foci in the cells and an increase in nuclear MSI1 foci and T22 in P301L tau iHEK, indicating a strong association between these two proteins. We observed an increment of MSI1 and tau nuclear foci in induced cells (Figure 4a). A double extraction analysis was also performed for MSI1 (green) and T22 (red) channels to quantify the number, diameter, and area ratios, of nuclear foci. As it can be observed in Figure 4a, there is an increment in nuclear foci size and number between induced and noninduced cells.

**Figure 5:**
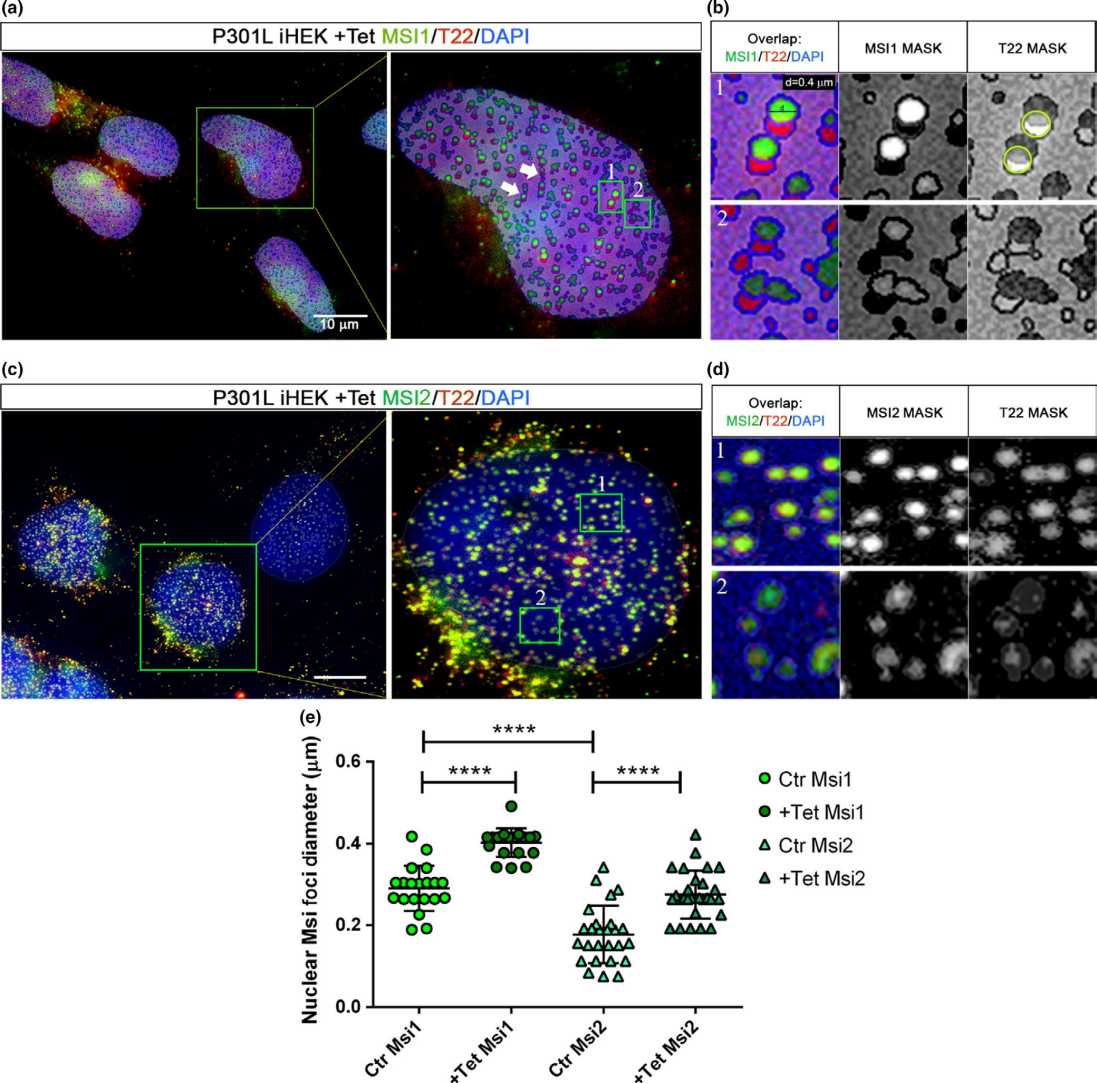
Overexpression of tau induced the formation of MSI nuclear macro‐structures. (a) Representative confocal image of P301L tau iHEK after double extraction of MSI1 (green) signal and T22 (red) signal, exclusively from the nuclei (target area). Selected nuclei (green square) and zoomed are represented. We observed different structures, evidenced by white arrows proto‐fibrils composed by alternate signal of MSI1 and T22. (b) *Inset 1* (green square 1 in a) zoomed showed the most common structure observed in the nuclei with MSI1 foci above T22 foci, with an overlap of 50% as we can observe in the T22 gray channel (green circle) where it is present the attenuate signal of T22 due to the overlap of MSI1. In B *Inset 2*, we observed that at a lower concentration, tau and MSI1 did not form an organized structure and they showed an irregular shape. (c) Representative confocal image of P301L tau iHEK after double extraction of MSI2 (green) signal and T22 (red) signal, from the nuclei. Selected nucleus (green square) and zoomed is represented. We observed, as well as for MSI1, different structures. The filaments have been observed (white arrows). (d) Inset 1 showed a complete overlap of the signals, and few elements showed comparable relationship as observed in MSI1, common feature is the irregular foci when the amount of signal is lower. (e) Graph of MSI nuclear foci diameter (µm^2^), showing an increment of MSI1 (****p < .0001) and MSI2 (****p < .0001) after Tet exposure in P301L tau iHEK. The graph showed also that the control MSI size is basically higher in MSI1 then MSI2 (****p < .0001) and that this difference is maintained after treatment (*p < *.0001). One‐way ANOVA and Tukey's multiple comparisons tests have been performed to demonstrate statistical significance

The diameter of nuclear MSI1 foci was measured, and a significant increment was found (Figure 4b) We observed a significant increase in co‐localized MSI1 and T22 numbers per nuclei (Figure 4c), coupled by an increase in their area ratio (Figure 4d) and nuclear area covered by the signals (Figure 4e). In addition, the sum of the MSI1 and T22 area ratios increased three times more in the number of nuclear foci from 6.70 ± 3.21% to 19.04 ± 1.86% of the nuclear area covered by these aggregates (Figure 4f). We also isolated nuclei from confocal imaging and evaluated the size and features of MSI1/T22 structures (Figure 5a). We observed structures with alternation of T22‐MSI foci (white arrows), and binary structures (partially overlapped) formed by MSI1 and T22 foci (Figure 5b—Inset 1). These structures represented the majority of structures revealed, but we also observed unorganized (anamorphic) structures with a low intensity signal (Figure 5b—Inset 2). We measured the diameter (ø) of such foci involved in these structures with an average of 0.4 µm (400 nm) MSI1 and T22 foci, and a major axis of 0.6 µm (600 nm) in P301L tau iHEK‐treated nuclei. The overlap was around 50% of the diameter between the foci. We also observed a significant increase in the nuclei of MSI2 foci density, with an increased area ratio. This was coupled with an increase in T22 signal as well (Figure 5c). Interestingly, all nuclear binary forms had the same orientation: on the bottom T22 signal and on the top MSI1 signal, and never the opposite. All these observations suggest that in the nucleoplasm, MSI1 and tau aggregates interact and form fine macromolecular structures with different grades of organization, from disorganized and low interaction shapes, to long pseudo‐filaments formed by the repetition of a binary system. Furthermore, to verify the overlap with T22, we selected the red channel to create a mask. The mask of the T22 channel showed that the T22 signal is also generating a circular shape (behind the green), indicating that tau aggregates may interact with MSI1 in these fine and highly organized structures. These structures have not been observed in the control nuclei. Furthermore, in Figure 5d, we observed MSI2 foci, but with a substantial difference in interaction by almost 100% overlapping with T22 oligomers, while maintaining the binary system (Figure 5d—Inset 1). Nuclear MSI2 and T22 oligomers were also present in disorganized and low signal foci (Figure 5d—Inset 2). We quantified the diameter of MSI1 and MSI2, and observed an increment in their diameter between control and treated cells. Notably, MSI1 foci were larger than MSI2 foci (Figure 5e).

### 3.5 Exogenous tau soluble aggregates form similar structures in tau HEK‐293 cells

To verify whether the oligomeric forms of tau induce nuclear changes in MSI expression and localization, which is previously shown with endogenous overexpression, we treated the HEK‐293 cells with recombinant 4R2N‐tauO labeled with Alexa Fluor 568 for 1 hr. After treatment with 0.0, 0.5, and 2.0 µM of tauO, the cells were fixed and imaged with a confocal microscope. For each condition, a representative image and plot profile (cytoplasm and nucleus) are shown. Lastly, quality control of TauO employed for these experiments was conducted and is shown in Figure S5.

**Figure 6:**
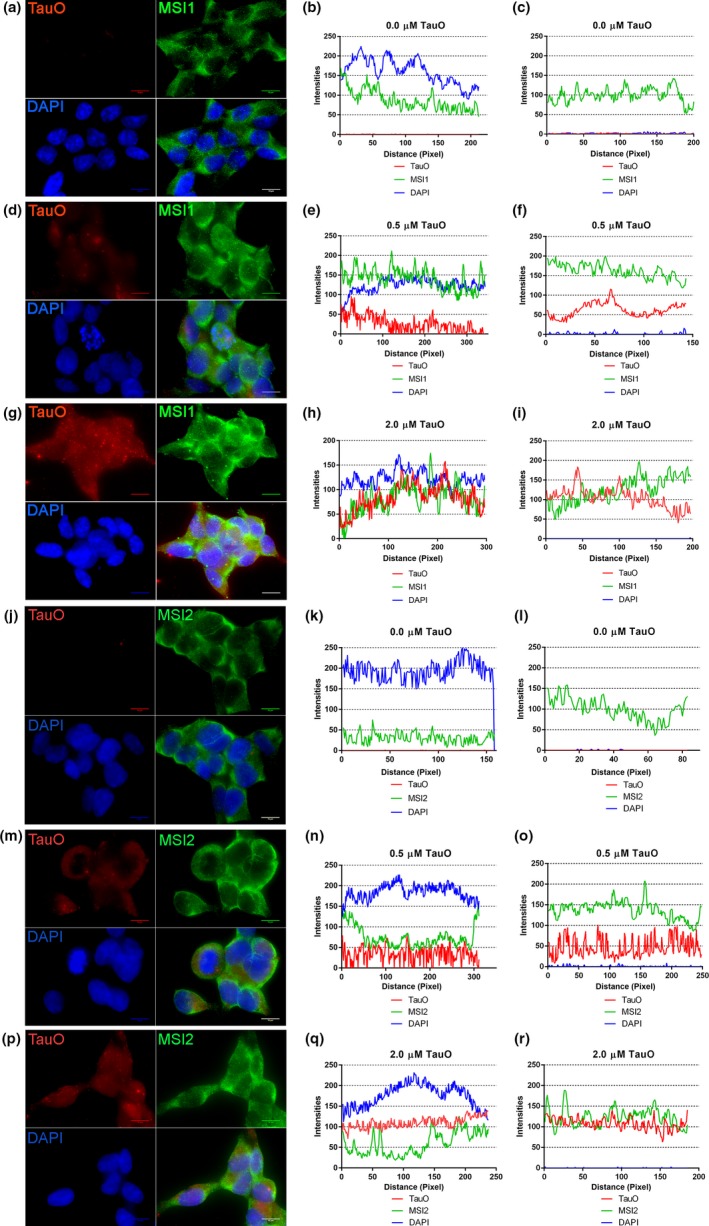
MSI1 and MSI2 co‐localized with TauO in nuclei and cytoplasm. MSI1 has been observed in control HEK293 in both compartments, prevalently in the cytoplasm (a). Plot profile of a representative control nuclei (b) and cytoplasm (c) showing higher intensities in the cytoplasm. When we incubate the cells with 0.5 µM of recombinant TauO, we observed an increment of nuclear and cytoplasmic signals (d). In particular, the nuclear plot profile (e) showed overlapping of MSI1 with DAPI indicating an increment of nuclear association between MSI1 and the nuclei, but no overlap was observed with TauO profiles (red lines) in both compartments, nuclei and cytoplasm (f). At the highest concentration (2 µM) of TauO (g), we observed signal comparable to the 0.5 cells, with the difference that in the nuclei all the profiles almost overlap (h) suggesting that at higher concentration MSI1 interact in the nuclei with TauO and that this association is now present also in the cytoplasm (i). All the images are taken with Objective 100× Nikon, oil immersion (scale bars: 10 µm), and the plot profiles (representative of 20 nuclei and 20 cytoplasm from different cells) are obtained with BX‐L Keyence Analyzer Software. MSI2 has been observed in control HEK293 in both compartments, and as previously shown for MSI1, it is prevalently in the cytoplasm (j). Plot profile of a representative control nuclei (k) and cytoplasm (l) showing higher intensities in the cytoplasm. When we incubate the cells with 0.5 µM of recombinant TauO, we observed an increment of nuclear and cytoplasmic signals (m). In particular, the nuclear plot profile (n) showed overlapping of MSI2 with TauO indicating a strong association between them in the nuclei, but no overlap was observed with TauO profiles (red lines) in the cytoplasm (o). At the highest concentration (2 µM) of TauO (p), we observed, in the nuclei, well separated profiles, except few points (q) suggesting that at higher concentration MSI2 interact at low level in the nuclei with TauO. In the cytoplasm, we observed a complete overlap of their profiles suggesting a strong association (r). All the images are taken with Objective 100× Nikon, oil immersion (scale bars: 10 µm), and the plot profiles (representative of 20 nuclei and 20 cytoplasm from different cells) have been obtained with BX‐L Keyence Analyzer Software

In Figure 6, we observed that MSI1 had increased signal in the nuclei at a treatment of 0.5 and 2.0 µM compared to the control (untreated) (Figure 6a,d,g). In the control, the MSI1 profile did not overlap with DAPI (Figure 6b), but at 0.5 µM, we observed a strong overlap between MSI1 and DAPI, indicating an increment in nuclear MSI1 with an improved proximity to the nuclei (Figure 6e). When treating the cells with 2.0 µM, the signal profiles from tauO and MSI1 overlapped strongly in the nuclei (Figure 6h). The MSI1 cytoplasm profile showed an increment at 0.5 and 2.0 µM as observed in the nuclei, but we observed an overlap of profiles only at 2.0 µM tauO treatment (Figure 6i) and no association at a 0.5 µM (Figure 6f). In control cells, MSI2 showed a low level in the nuclei (Figure 6j, k), and a higher signal was seen in the cytoplasm (Figure 6l). In cells treated with 0.5 µM tauO, we observed a nuclear increment of MSI2 signal (Figure 6m, n) as well as in the cytoplasm (Figure 6o). At 2.0 µM (Figure 6p), we observed a strong increment of T22 signal in the nuclei (Figure 6q), and comparable T22 level in the cytoplasm (Figure 6r).

In summary, we observed that tauO induced changes in localization and the fluorescent pattern of MSI proteins in a concentration‐dependent manner. In particular, MSI1 showed interpolation with tauO at higher concentrations in the cytoplasm and nuclei (Figure S5), but not at low concentration of 0.5 µM tauO. In contrast, MSI2 showed co‐localization in cytoplasm at concentration of 2.0 µM (Figure S5). However, in the nuclei, such interaction was observed at the lower concentrations (Figure S5). In general, we observed that MSI1, unlike MSI2, interacts with tauO at elevated concentrations in the nuclear and cytoplasmic compartments. All these results suggest that soluble TauO interact with MSI. At a higher concentration, tauO co‐localizes in the cytoplasm and nuclei with MSI1, while only in the cytoplasm with MSI2. These observations also suggest that MSI2 mediates the interaction at lower concentrations, whereas, at higher oligomeric concentrations, the majority of tau strongly associates with MSI1 in both compartments. It is interesting that at 0.5 µM, MSI1 is associated with the DAPI profile (not observed for MSI2), suggesting that tauO induces more nuclear association (proximity) of MSI1 at a low concentration through an indirect pathway that channels MSI1 into the nuclei.

### 3.6 Nucleus–cytoplasm shuttling of MSI1 and tau is Importin‐β mediated

Previous observations suggest that the increment of MSI nuclear signal is due to tau. To establish which mechanism is involved in the nuclear translocation, we inhibited Importin‐β with a specific inhibitor: Importazole (IPZ; Soderholm et al., 2011). The iHEK cells were treated with IPZ at different concentrations (10, 20 and 40 µM) for 12 hr with/without Tet induction (24 hr).

**Figure 7:**
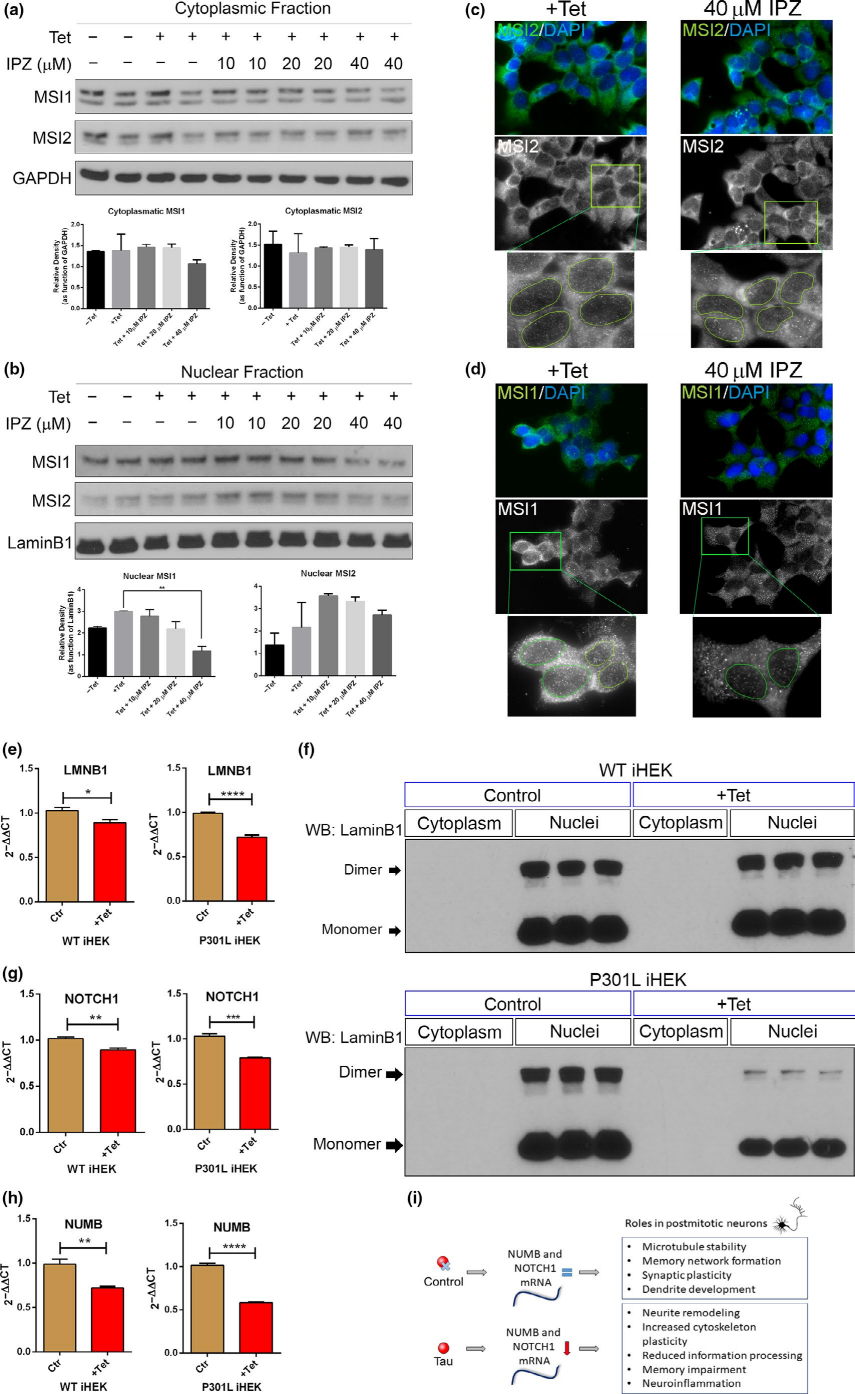
Nuclear Import of MSI and tau and LaminB1 down‐regulation. (a) Western blot of cytoplasmic fraction from P301L tau iHEK showed no significant changes in MSI1 and MSI2 after treatment with IPZ. (b) Western blot of P301L tau iHEK nuclear fraction showed significant decrement of MSI1 at 40 Mm of IPZ (**p < .05), and MSI2 did not show a significant reduction. (c) Representative confocal images of MSI2 immunofluorescence of +Tet and 40 Mm IPZ cells; MSI2 is represented in gray and three zoomed detail (green square) showing no big difference on nuclear signal from MSI2. (d) Representative confocal images of MSI1 (green) immunofluorescence of +Tet and 40 µM IPZ cells, MSI1 is represented in gray and three zoomed detail area (green square) showing a marked difference on nuclear MSI1 signal, with a reduction of signal in cells treated with 40 µM of IPZ. One‐way ANOVA and Dunnett's multiple comparisons tests have been performed to demonstrate statistical significance. (e) Overexpression of WT tau and P301L tau down‐regulates LMNB1 gene expression. The decrement is significant in WT iHEK (*p = .0102) but it is more conspicuous in P301L tau iHEK (****p < .0001). (f) The difference in LMNB1 gene expression has been confirmed with Western blot using LaminB1‐specific antibody. In WT tau iHEK, there was not visible difference between the nuclear fraction in control and induced cells. This became evident in P301L tau iHEK nuclear extracts where LaminB1 monomer and dimer level decreased after induction. To link a known pathway to MSI activity, we verified gene expression of NUMB and NOTCH1, downstream effectors of MSI. NOTCH1 expression decreased in WT iHEK (**p = .0012) and in P301L tau iHEK (***p = .0001) (g). These trends have been observed also for NUMB in WT (**p = .0015) and P301L tau iHEK (****p < .0001) (h). In (i), it is shown how expression of tau through MSI proteins modulate gene expression and function of NUMB/NOTHC1 pathway and the effects, potentially, we can observe in postmitotic neurons. An active pathway, play a role in microtubule stability and synaptic plasticity and it has been shown to play an important role in dendrite development too. However, when this pathway is down‐regulated neurite remodeling, cytoskeleton remodeling, reduced information processing, and memory and also neuro‐inflammation have been observed

As shown in Figure 7a, we did not observe any significant differences in cytoplasmic MSI level between controls and treated cells. The only significant decrease observed was in the nuclear fraction of MSI1 in cells treated with 40 µM of IPZ (+Tet vs. Tet + 40 µM IPZ, **p < .05). No difference in the nuclear fraction was observed for MSI2 at the same concentration (Figure 7b). Representative fluorescence images of +Tet cells treated with 40 µM and stained for MSI2 showed no difference in their nuclear signals (Figure 7c), while MSI1 in the same treated group showed a strong nuclear reduction (Figure 7d). We also evaluated cytoplasmic and nuclear tau levels under the same conditions, and observed a strong reduction of nuclear monomeric tau in cells treated with 20 and 40 µM (Figure S6). These combined evidence indicate that MSI1 and tau are shuttled from the cytoplasm to the nuclei via an Importin‐β‐dependent mechanism, whereas MSI2 is likely to use another nuclear transporter. Nucleophosmin antibody has been used as positive control (Figure S6).

### 3.7 Tau mediates nuclear lamina dysfunction

We verified the gene expression of LMNB1 (coding for LaminB1 protein) with RT–qPCR and observed a reduction of its gene expression in WT and P301L tau cells induced with Tet (Figure 7e). We observed a remarkable reduction of LaminB1 protein (monomer and dimer) levels by Western blot analysis in iHEK P301L tau cells. These results suggest that tau controls one of the major components of nuclear lamina at different levels. Such decrease in gene expression was remarkable in P301L tau cells. However, large differences were not observed in the WT LaminB1 protein levels (Figure 7f—top panel). It has been shown that tau modulates LaminB1 expression and chromatin relaxation (Frost et al., 2016, 2014). In this scenario, we found that the mutant tau form has the strongest effect, as opposed to WT tau species. This strong reduction in LaminB1 can mediate nuclear dysfunction, impairing structural and functional properties. To verify possible downstream effects of MSI/tau aggregation, we evaluated the mRNA level of one of the identified MSI mRNA targets, Numb, that is inhibited by MSI (Jadhav et al., 2016; Sheng et al., 2017). Numb protein normally inhibits the activation of Notch receptors (Giebel & Wodarz, 2012). Notch deregulation is involved in many neurodegenerative diseases and brain disorders (Zhang, Engler, & Taylor, 2018). MSI1 is known to bind to the 3′‐untranslated region (UTR) of several target mRNAs and to regulate these genes post‐transcriptionally, in particular, those encoding Numb and p21 (Battelli, Nikopoulos, Mitchell, & Verdi, 2006; Imai et al., 2001). Numb binds and inhibits the NICD fragment generated from the γ‐secretase cleavage of the Notch1 receptor. Active NICD translocates to the nucleus and activates the transcription of target genes. In our study, NOTCH1mRNA expression was strongly compromised (Figure 7g), as well as NUMB (Figure 7h). Given this context, we observed that MSI expression was up‐regulated, as gene expression of NUMB and NOTCH1 was strongly down‐regulated in the presence of WT and P301L‐mutated tau. These results suggest that tau modulates the Numb/Notch1 pathway. Possible effects of Notch1 down‐regulation are summarized in Figure 7i, including multiple neuronal dysfunctions from the reduced information processing to memory impairment and neuro‐inflammation, all of them being hallmarks of AD pathology.

### 3.8 MSI proteins interact with tau in the cytoplasm and in the nuclei of cells

**Figure 8:**
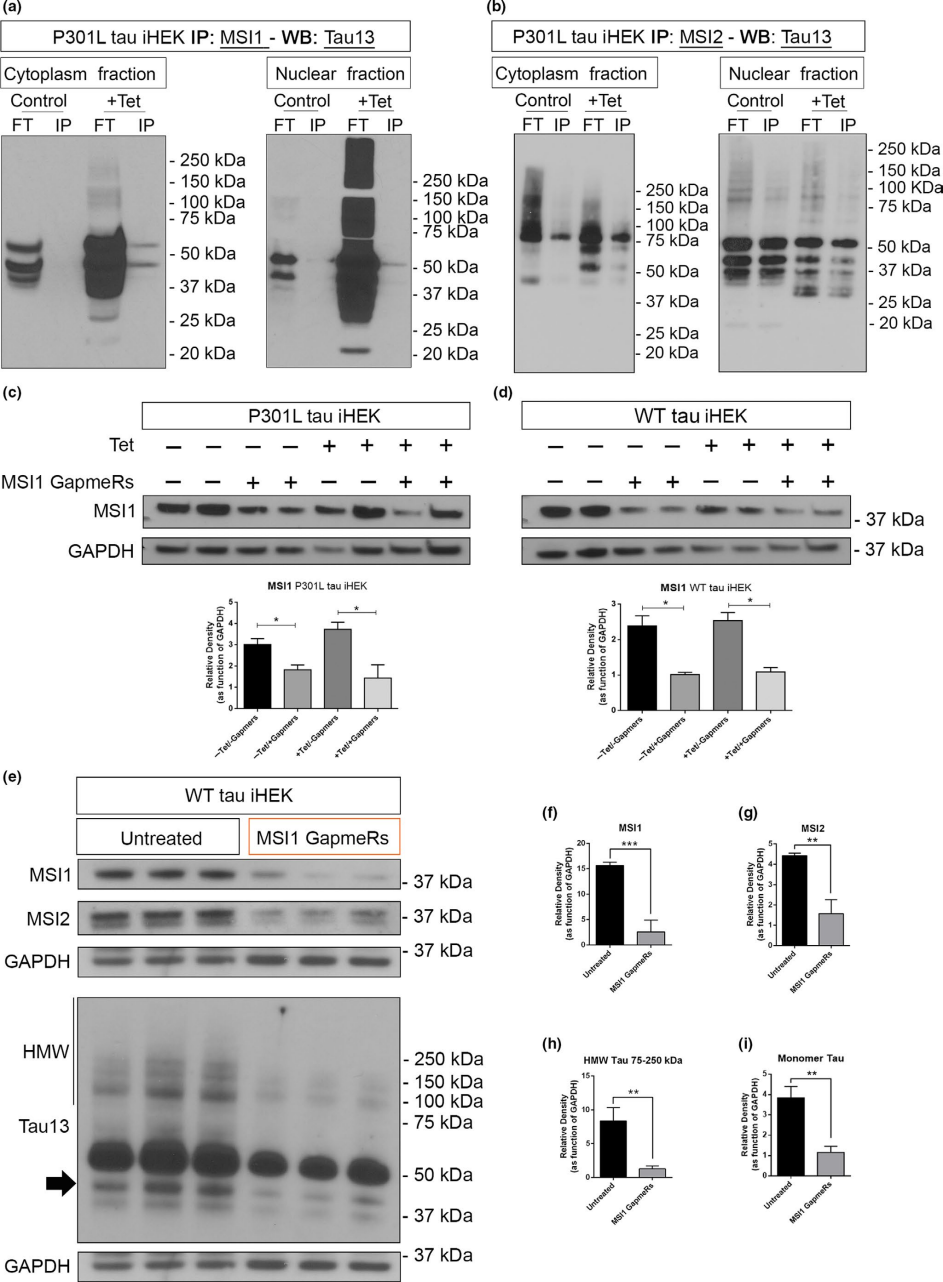
Interaction of tau with MSI proteins and MSI1 silencing effect on tau levels. (a) Western blot of tau13 in MSI1 IP cytoplasm and nuclear fractions from P301L tau iHEK. (b) Western blot of tau13 in MSI2 IP cytoplasm and nuclear fractions from P301L tau iHEK. (c) Western blot of MSI1 from total lysate of P301L tau iHEK and relative quantification. (d) Western blot of MSI1 from total lysate of WT tau iHEK and relative quantification. (e) Western blot of MSI1, MSI2, and Tau13 in untreated and silenced WT tau iHEK total lysates. (f) MSI1 quantification. (g) MSI2 quantification. (h–i) HMW (75–250 kDa) tau and monomeric form (black arrow) quantification. Bar Graphs are used to show quantification of relative density as function of GAPDH. Student's *t* test has been use to determine statistical significance. Error bars represent *SD*

Immunoprecipitation assay was performed to show interaction of MSI proteins with tau. Antibodies for MSI1 and MSI2 were used for IP from nuclear and cytoplasmic fractions followed by Western blotting with Tau13 antibody. Both MSI proteins were able to bind to different tau species as detected in the Western analysis. In particular, we observed monomeric form of tau in the cytoplasmic and nuclear fractions IP’d with MSI1 antibody after Tet induction (Figure 8a). In MSI2 IP’d cytoplasmic fraction, monomeric tau forms and HMW tau form between 70 and 100 kDa were noticed, while the MSI2 IP’d nuclear fraction mostly contained monomeric and cleaved forms of tau (Figure 8b). Interestingly, we observed different forms of tau between MSI1 and MSI2 IP’d fractions, indicating that different interactions between MSI and tau species can occur in different cell compartments (Figure 8a‐b). iHEK cell model provides the possibility to modulate tau expression. To study the possible effect of MSI1 on tau, we performed a transient and stable knockdown with Gapmers technology. We confirmed significant reduction in MSI1 level in both P301L and WT tau iHEK cells (Figure 8c,d, respectively). In WT tau iHEK, we confirmed the silencing of MSI1 and observed a similar pattern of reduction in MSI2 and tau levels (Figure 8e–i). In particular, HMW and monomeric tau forms were significantly reduced in silenced cells as evident from Western blot and relative quantifications (Figure 8e,h,i). Additionally, MSI2 showed significant reduction (Figure 8g). These observations suggest that MSI1 could control tau and MSI2 expression (Figure S7). Efficiency of Gapmers internalization and Tet effects on silencing are shown in Figure S7.

### 3.9 Tau oligomers affect MSI1 cellular localization in primary neurons

**Figure 9:**
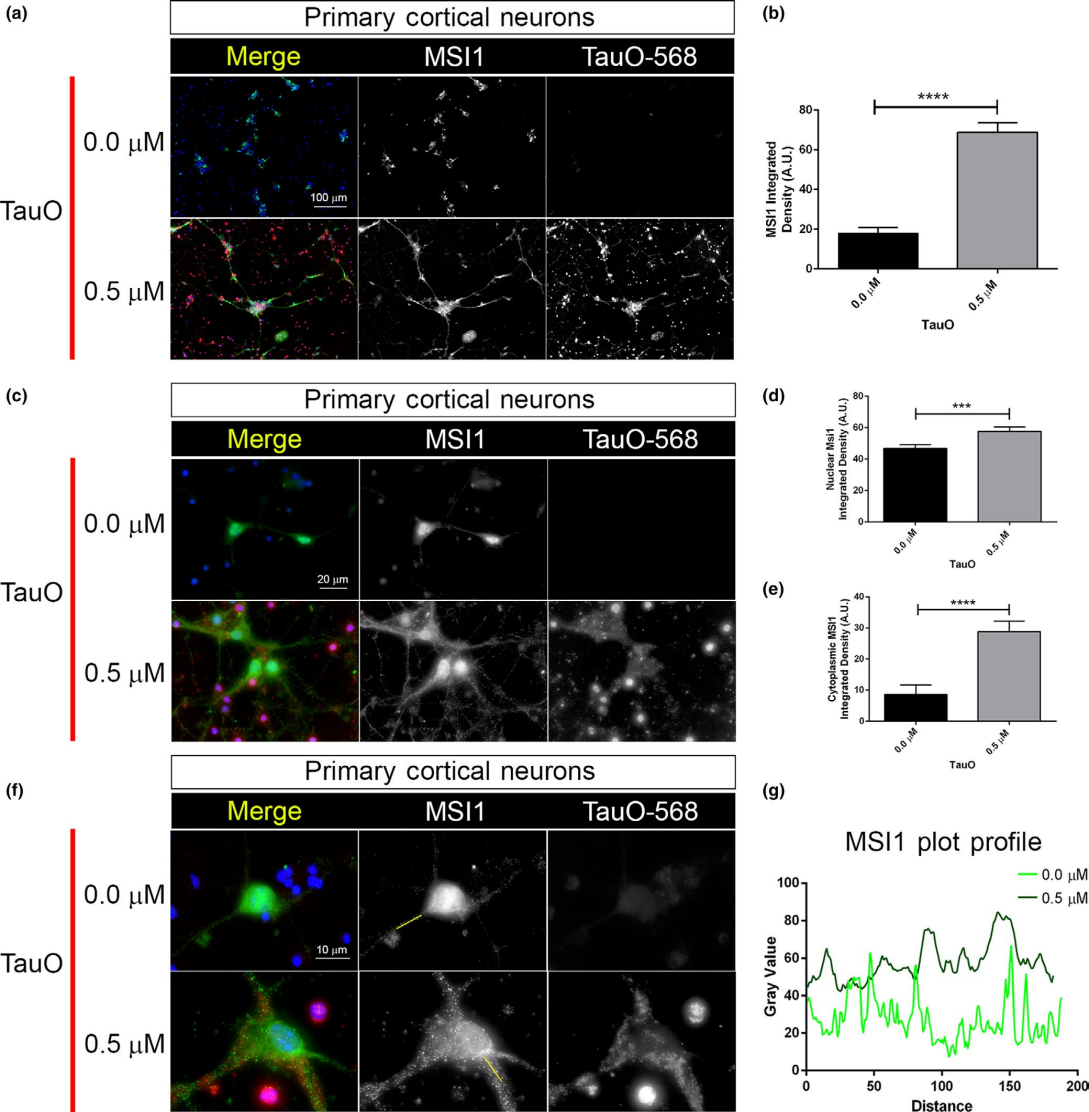
Tau oligomers affect MSI1 cellular localization and accumulation in primary neurons. (a) Representative confocal images of untreated and treated (0.5 µM tauO) primary cortical neurons. Images are represented by merge, MSI1 (green channel) and tauO‐568 (red channel); both channels are represented in gray for a better contrast (magnification: 20×, white scale bar: 100 µm). (b) MSI1 integrated density quantification in untreated and tauO‐treated neurons (****p < .0001). (c) Representative confocal images of untreated and treated (0.5 µM tauO) primary cortical neurons. Images are represented by merge, MSI1 (green channel) and tauO‐568 (red channel); both channels are represented in gray (magnification: 100×, white scale bar: 20 µm). (d–e) MSI1 integrated density quantification, respectively, in nuclei and cytoplasm (0.0 µM vs. 0.5 µM, ***p = .0002 and ****p < .0001, respectively). (f) Representative confocal images of untreated and treated (0.5 µM tauO) primary cortical neurons. Images are represented by merge, MSI1 (green channel) and tauO‐568 (red channel); both channels are represented in gray (magnification: 100×, optical zoom: 3× white scale bar: 10 µm). (g) Intensity plot profiles from representative axonal MSI1 signals. Light green line represents untreated neurons segment, while dark green line represents tauO‐treated neurons segment. Represented segments are highlighted in solid green line in (f) MSI1 channel. Nuclei are with DAPI, and bar graph is used to show integrated density measurements (error bars represent *SD*)

To validate our observations in a neuronal system, we isolated and cultured primary cortical neurons from embryonic P301L mice brain. Specifically, after 10 days in culture, we treated primary neurons with 0.5 µM of labeled tauO for 1 hr. We reproduced the same condition that was used for P301L tau iHEK cells. After 1 hr of incubation, neurons were fixed and imaged with a confocal microscope. We observed in untreated neurons a large amount of MSI1 in the nuclei, while in tauO exposed neurons, we observed a general increment of MSI1 signal in the cytoplasm of cell body and in the neuronal projections (Figure 9a). This intensity difference has been measured confirming a significant increment of MSI1 signal in neurons after incubation with tauO (Figure 9b). The difference in MSI1 distribution became evident in higher magnification images (100×, Figure 9c), where we observed large amount of MSI1 in the projections of tauO exposed neurons compared to the untreated cells. We quantified and compared MSI1 signal from nuclei and cytoplasm, confirming an increment of MSI1 in both compartments (Figure 9d–e). To better describe the distribution and size of MSI1 foci, we also collected zoomed images of single neuron where we observed that in treated cell MSI1 forms larger foci than the untreated cells (Figure 9f). This observation was confirmed by the comparison of MSI1 profile plot in Figure 9g, In untreated neurons, we observed a profile with several spikes indicating the presence of numerous and little MSI1 foci. On other hand, we observed an higher intensity of MSI1 signal but also big and large picks in the neurons treated with tauO. Taken together, these observations indicate that tauO induced MSI1 accumulation in cytoplasm and nuclei of the neurons, thereby changing cellular localization of this RNA‐binding protein. Thus, comparable results from both iHEK cells and primary cortical neurons support the hypothesis of a possible role and effect of MSI proteins in the pathogenesis of neurodegenerative diseases.

## 4 DISCUSSION

We previously showed, in AD brains, the co‐localization and interaction of MSI with tau (Sengupta et al., 2018). Although our previous study suggests that MSI and tau interact in the neuronal cytoplasm and nuclei, the exact mechanism driving their initial accumulation and toxicity remains unclear.

In this report, we demonstrate for the first time that tau regulates MSI by promoting tau/MSI nuclear localization and accumulation in vitro. In particular, we have identified such interactions in the cytoplasm and nuclei of the cells. Our observation here is the extension of previous studies, indicating that altered cellular distribution of tau and RBPs contributes to neurodegeneration (Barmada et al., 2010; Fernandez‐Nogales et al., 2014). Indeed, several studies suggest that mis‐localization of tau as a result of mutations, post‐translational modifications, or overexpression also contributes to neurodegeneration (Frandemiche et al., 2014). Our findings suggest that MSI proteins drive the nuclear accumulation of tau coupled with an alteration of the nuclei. This provides new mechanistic insights into the steps that lead to the pathogenicity of tau.

We also observed the formation of HMW tau and MSI aggregates in the cytoplasm and nuclei of the cells, indicating possible compartment‐specific functions that were not investigated here. Endogenous tau (WT and P301L forms) influences the MSI protein levels and localization; in particular, P301L mutant tau majorly influences MSI expression and aggregation in the cytoplasm and nucleoplasm. Interestingly, in this study we observed an abnormal accumulation of HMW soluble aggregates of MSI proteins. MSI proteins enhanced tau nuclear translocation with an increase in perinuclear foci density and size. The molecular mechanism through which MSI stabilizes and mediates the nuclear localization of tau is not yet elucidated. Defects of nucleocytoplasmic transport contribute critically to the pathology of several neurodegenerative diseases (Fahrenkrog & Harel, 2018). The main perturbations are associated with displacement of nuclear transport and nuclear pore complex, as well as mis‐localization and aggregation of RBPs (Fahrenkrog & Harel, 2018). The importin family is one of the most important transporter families in eukaryotic cells, as many cargos bind nuclear transporter Importin‐β (Lott & Cingolani, 2011; Nuovo et al., 2018). We observed that MSI1 and tau are imported into the nuclei by Importin‐β while MSI2 is not. It is possible that MSI generally mediates their effects on tau nuclear import indirectly through an un‐identified mediator. Other transporters, not investigated here, may likely regulate MSI2 nuclear import. We also observed the presence of nuclear complexes between MSI and TauO along with increments of MSI foci density and size in the nuclei. A partial overlap of sub‐rounded shaped structures between MSI1 and tau, and completely overlapped foci between MSI2 and tau were noticed. Moreover, we show that with the two MSI proteins result in two different nuclear macro‐structures, suggesting potential polymorphism in the complexes formed.

Findings obtained from iHEK cell lines are supported by the observation of comparable results using mouse primary cortical neurons. As expected, we observed that tauO also induced changes in MSI1 accumulation and cellular localization in primary cortical neurons, thus pointing out to a crucial partnership between toxic tau species and MSI proteins. Further investigations will provide significant information on such interaction.

All these evidence (summarized as a model in the graphical abstract) suggest that MSI mediates tau aggregation, and vice versa, in nuclear and cytoplasmic compartments. Specifically, tau/MSI complexes cause destabilization of the nuclei through the inhibition of LaminB1 expression and disrupt the nuclear architecture. An understanding of the precise mechanisms that affect MSI accumulation as well as tau nuclear localization in human tissue will provide additional context for the understanding of disease pathogenesis. We present two potential mechanisms through which deregulated MSI activities could promote neurodegeneration. The movement of tau to the nucleus induces nuclear instability and/or allows for a toxic gain of function in the nuclei. Furthermore, the accumulation of tau in the cytoplasm dysregulates the NUMB/NOTCH1 pathway. It is increasingly important to identify shared modifiers and regulatory mechanisms of early stages of disease‐causing proteins, both to understand the AD pathogenesis and to find effective candidates for therapeutic interventions. Future studies will dissect such structures with genetically engineered constructs that permit monitoring the domains and/or sequences involved in these interactions.

## CONFLICT OF INTEREST

None declared.

## AUTHOR CONTRIBUTIONS

M.M. and R.K. involved in conceptualization; M.M., U.S., and R.K. performed methodology; M.M., S.M., N.P., N.B., A.E., U.S., and R.K. investigated the study; M.M. wrote—original draft; all authors wrote—review and editing; R.K. carried out funding acquisition; R.K. provided resources; M.M. and R.K. supervised the study.

## Data Availability

The datasets used and/or analyzed in this current study are available from the corresponding author.
