# Supplementary figures and images for: Tau oligomers mediate aggregation of RNA‐binding proteins Musashi1 and Musashi2 inducing Lamin alteration
Source: Aging Cell. 2019 Sep 18;18(6):e13035. doi: 10.1111/acel.13035 (PMC6826126; doi:10.1111/acel.13035)

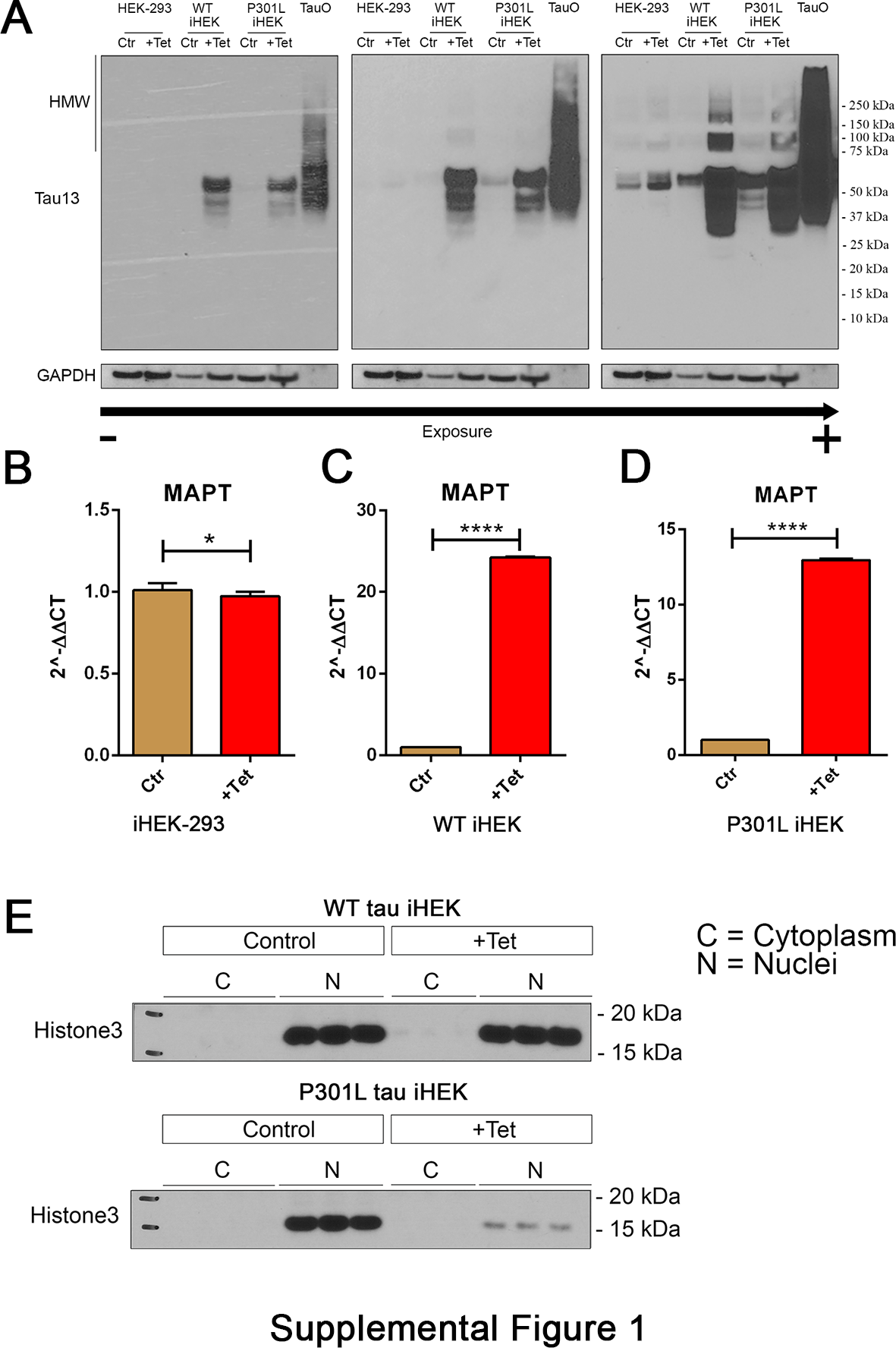

Supplement: Supplementary file 1 [file ACEL-18-e13035-s001.tif]

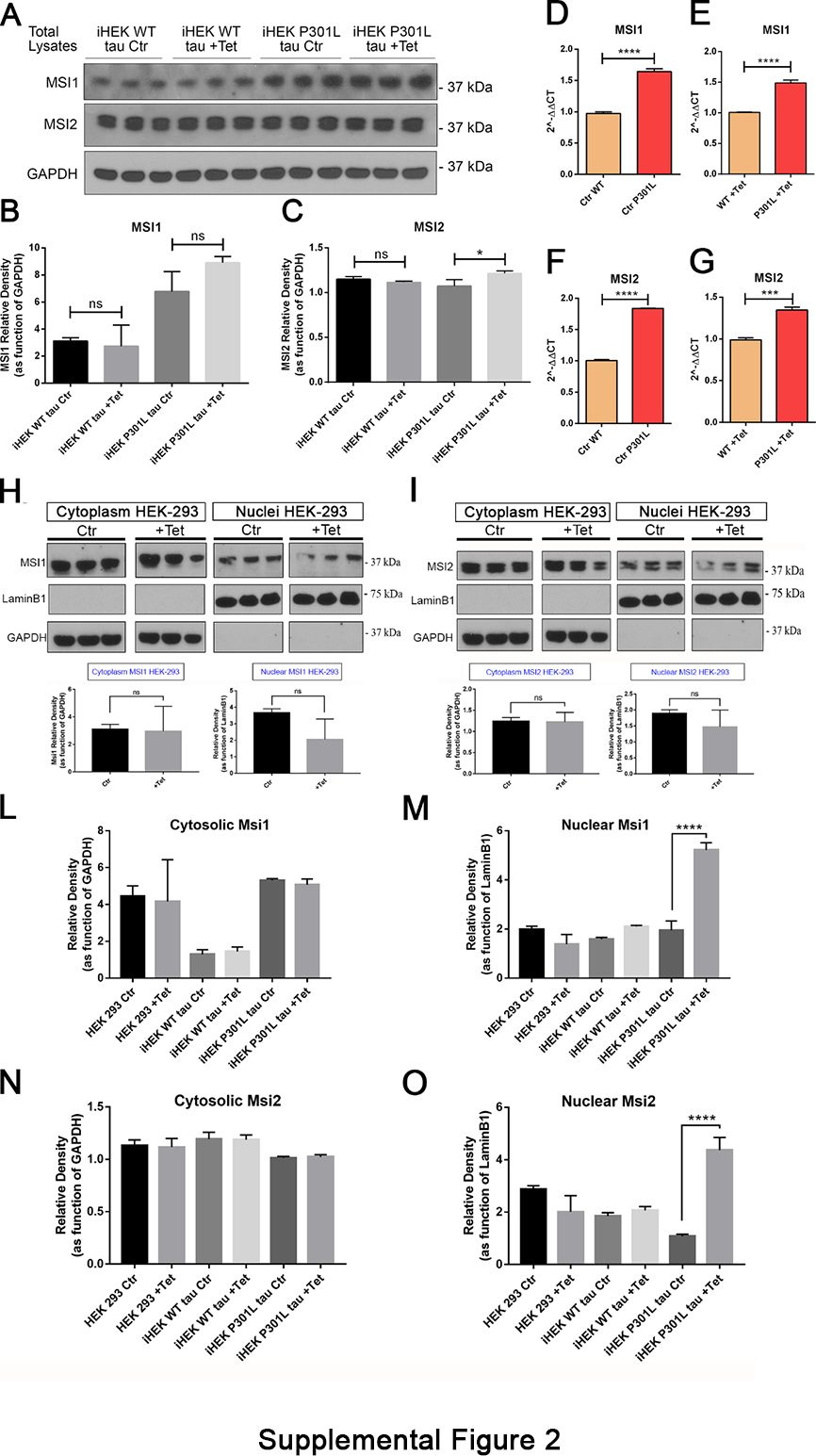

Supplement: Supplementary file 2 [file ACEL-18-e13035-s002.tif]

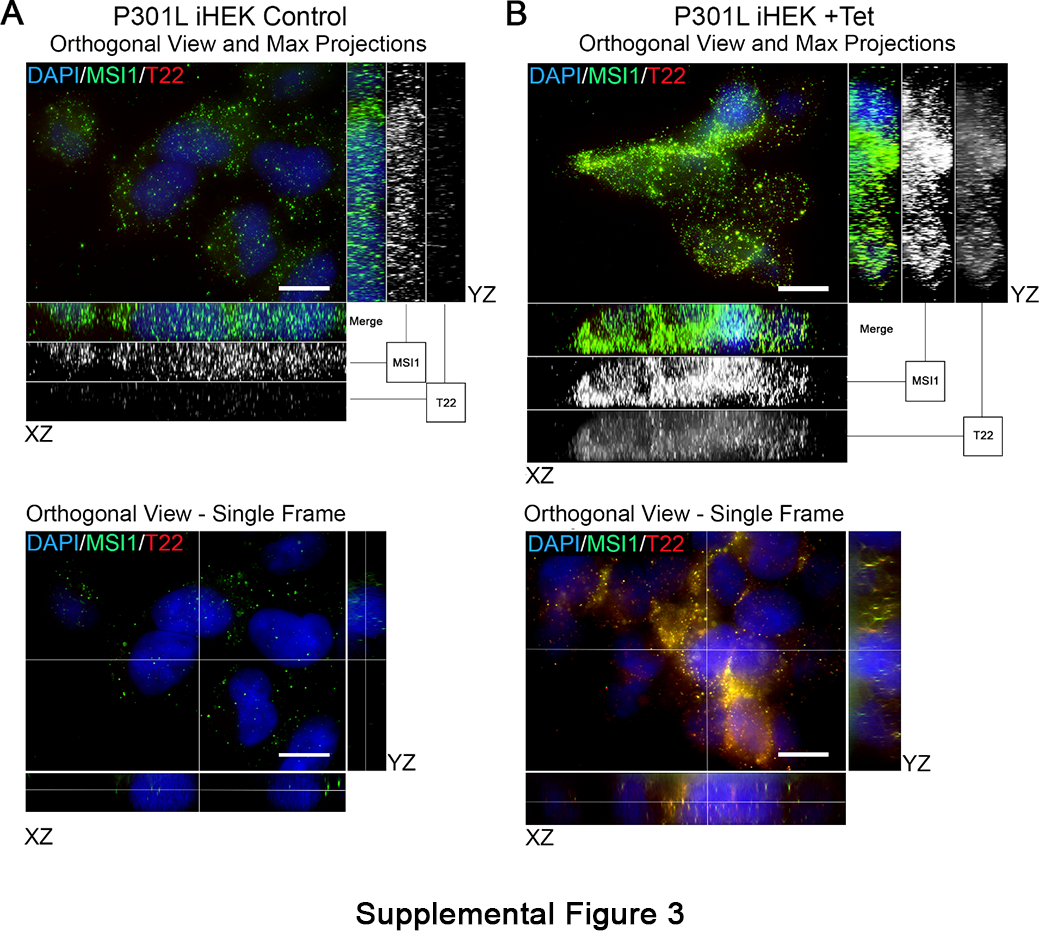

Supplement: Supplementary file 3 [file ACEL-18-e13035-s003.tif]

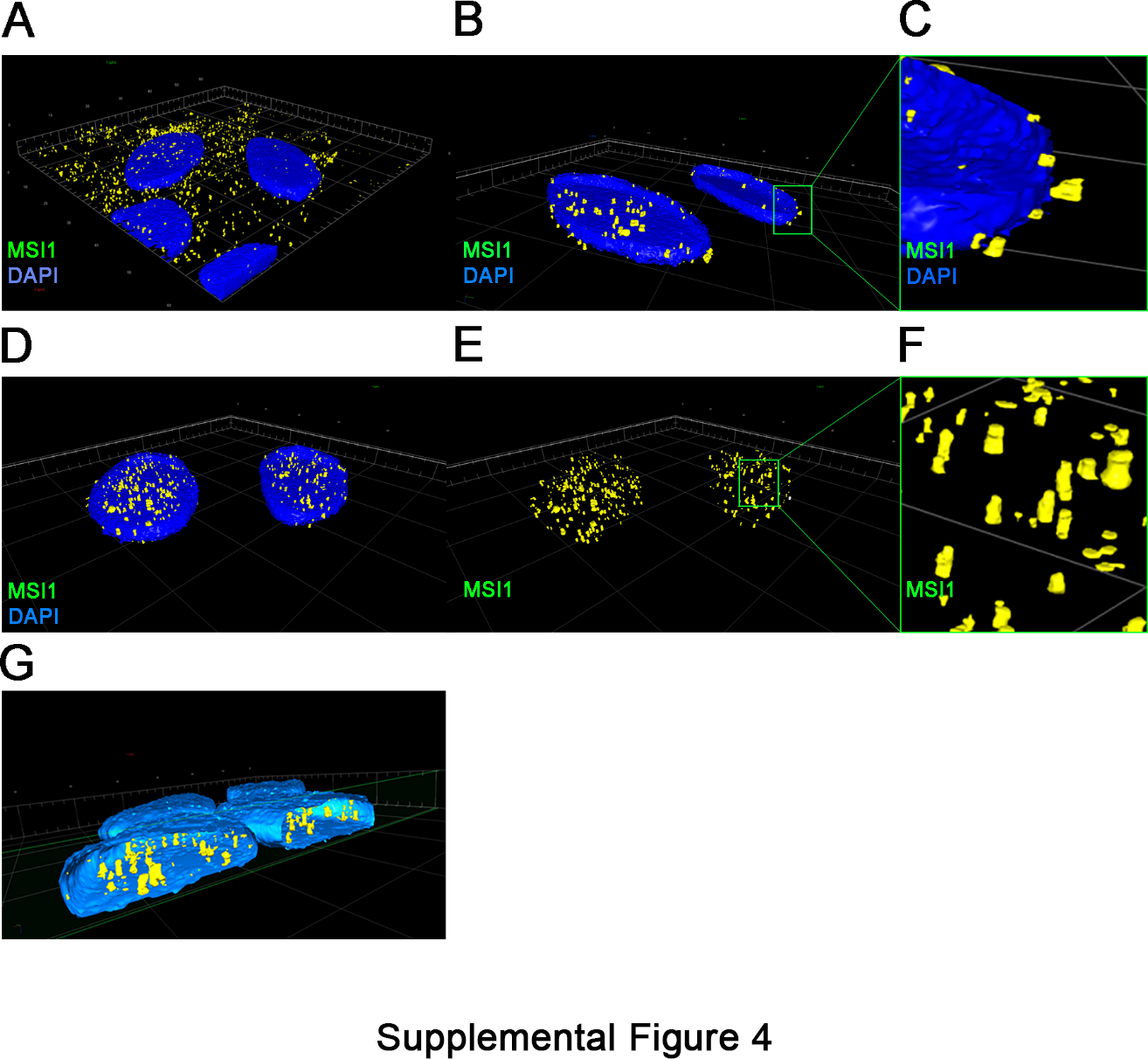

Supplement: Supplementary file 4 [file ACEL-18-e13035-s004.tif]

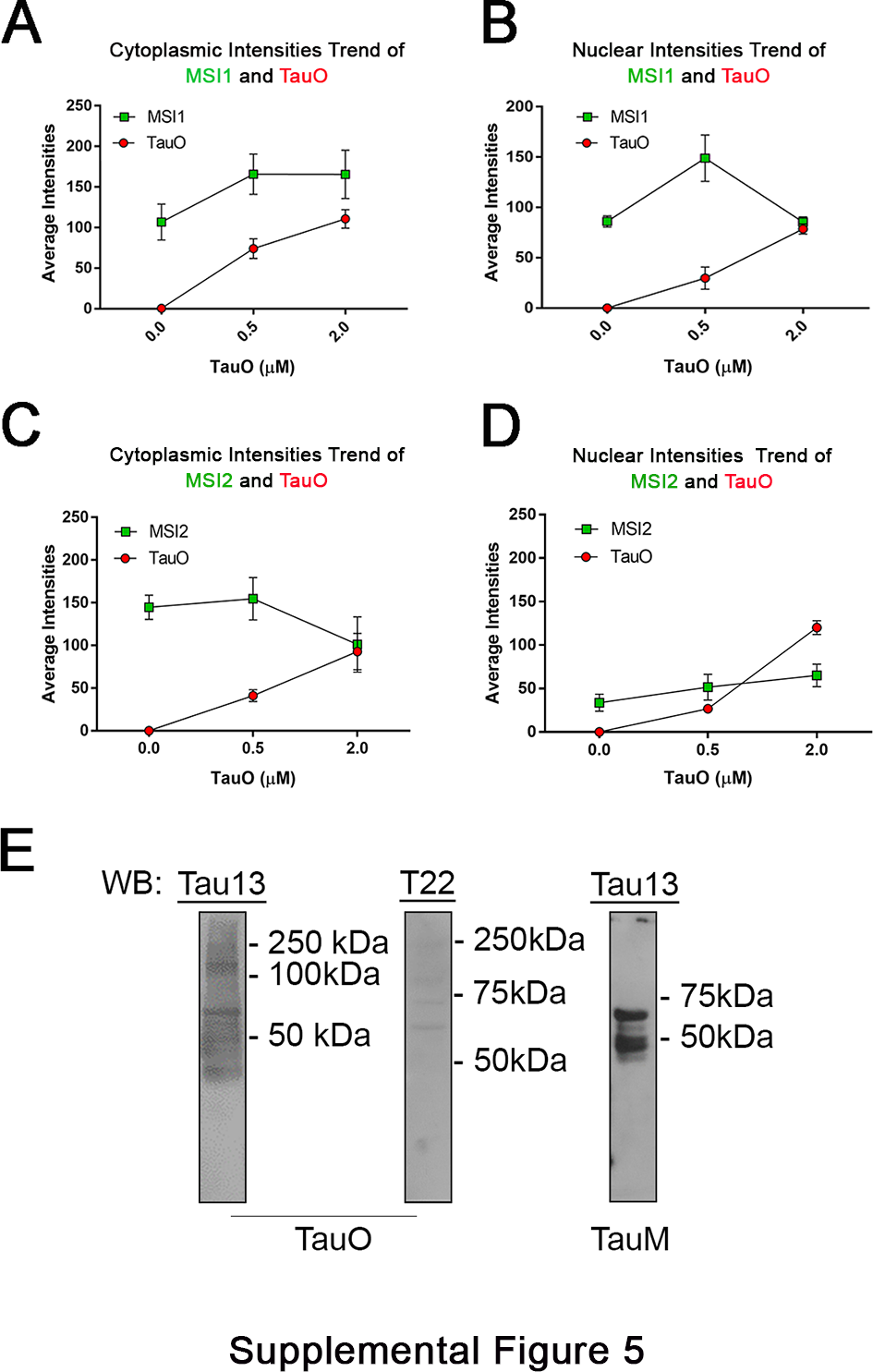

Supplement: Supplementary file 5 [file ACEL-18-e13035-s005.tif]

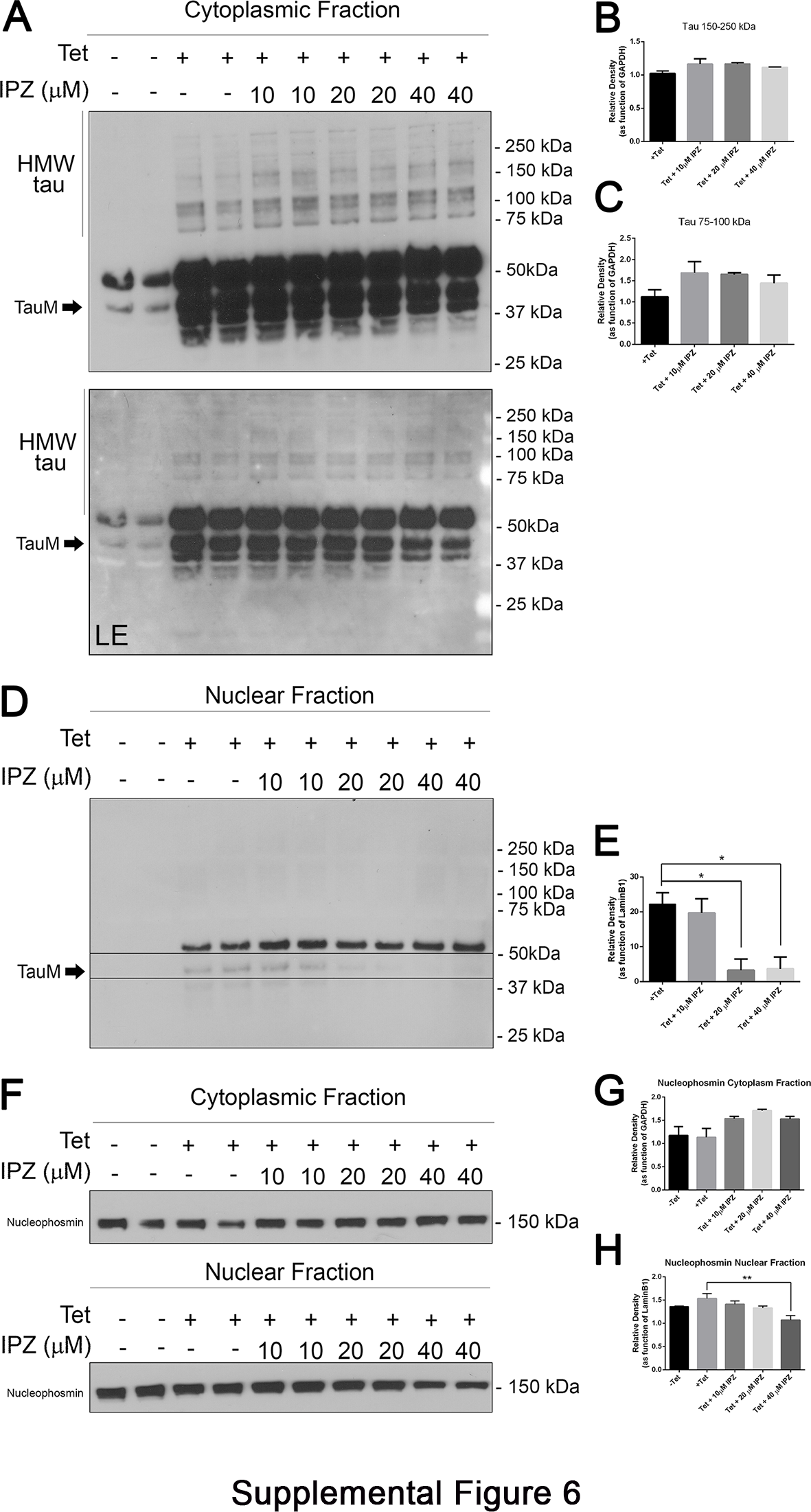

Supplement: Supplementary file 6 [file ACEL-18-e13035-s006.tif]

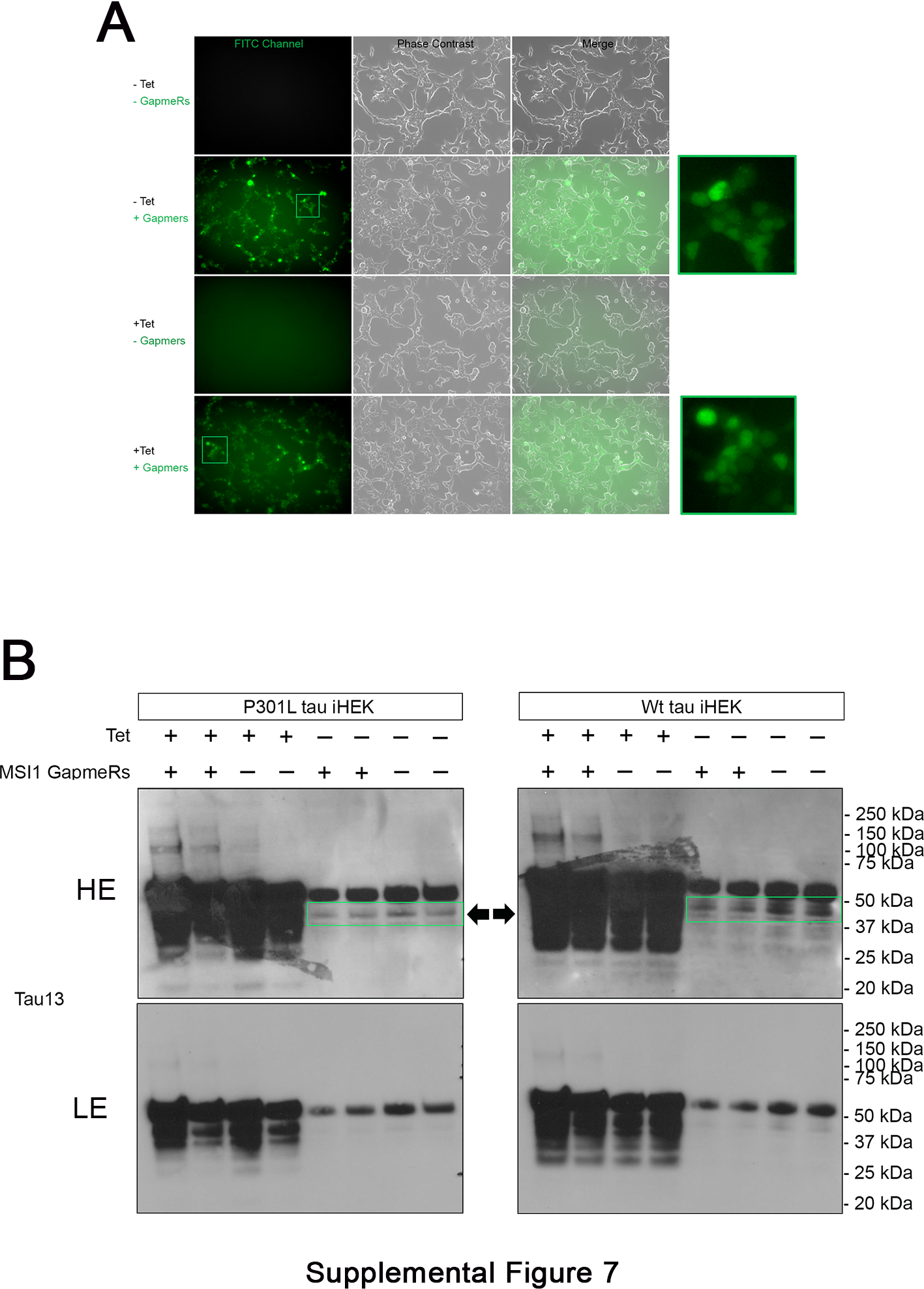

Supplement: Supplementary file 7 [file ACEL-18-e13035-s007.tif]
