# Supplementary material for: Tau oligomers mediate aggregation of RNA‐binding proteins Musashi1 and Musashi2 inducing Lamin alteration
Source: Aging Cell. 2019 Sep 18;18(6):e13035. doi: 10.1111/acel.13035 (PMC6826126; doi:10.1111/acel.13035)
Supplement: Supplementary file 8 [file ACEL-18-e13035-s008.docx]

**Supplemental Materials**

**List of RT-qPCR primers:**

**MAPT primers:**

Name: MAPT-2238F 25 bases TM = 58C

Sequence: GCAGTGTGCAAATAGTCTACAAACC

Name: MAPT-2351R 22 bases TM = 59C

Sequence: CAGATTTTACTTCCACCTGGCC

Amplicon = 114bp

**MSI2 primers:**

Name: MSI2-1111F 22 bases TM = 60C

Sequence: GCATAGCTGGACCTTTGATTGC

Name: MSI2-1244R 22 bases TM = 59C

Sequence: CAAAGGGCCACTCAGAAACTTC

Amplicon = 134bp

**MSI1 primers:**

Name: MSI1-486F 17 bases TM = 59C

Sequence: GGGAAGGTGGACGACGC

Name: MSI1-587R 23 bases TM = 60C

Sequence: CACTTTCTCCACGATGTCCTCAC

Amplicon = 102bp

**LMNB1 Primers**

Name: LMNB1-2356F 21 bases TM = 58C

Sequence: GAAGGCAGGCCAGACTGTTAC

Name: LMNB1-2472R 21 bases TM = 59C

Sequence: ACCTTCACATCTTCGCCAGTG

Amplicon = 117bp

**NOTE:** Assay detects variants 1 & 2.

**NOTCH1 Primers**

Name: NOTCH1-6402F 20 bases TM = 58C

Sequence: TCCTGAAGAACGGGGCTAAC

Name: NOTCH1-6526R 18 bases TM = 59C

Sequence: GTCCCGGTTGGCAAAGTG

Amplicon = 125bp

**NUMB Primers**

Name: NUMB-434F 22 bases TM = 60C

Sequence: CTTCCCGGTTAAGTACCTTGGC

Name: NUMB-564R 24 bases TM = 59C

Sequence: TCTTTCCAGTTTTTCCAAAGAAGC

Amplicon = 131bp

**NOTE:** Assay detects variants 1, 2 & 5.
