# Supplementary material for: Tau oligomers mediate aggregation of RNA‐binding proteins Musashi1 and Musashi2 inducing Lamin alteration
Source: Aging Cell. 2019 Sep 18;18(6):e13035. doi: 10.1111/acel.13035 (PMC6826126; doi:10.1111/acel.13035)
Supplement: Supplementary file 9 [file ACEL-18-e13035-s009.docx]

Table S1 List of antibodies and reagents

| **REAGENT or RESOURCE** | **SOURCE** | **IDENTIFIER** |
| --- | --- | --- |
| **Antibodies** | | |
| Tau-13 | BioLegend | # MMS-520R |
| T22 | In-house |  |
| Musashi1 | Abcam (ICC/IF, WB) | ab52865 |
| Musashi2 | Abcam (IP, ICC/IF,WB) | ab76148 |
| GAPDH | Abcam | Ab9485 |
| LaminB1 | Abcam | ab133741 |
| Histone3 | Abcam | ab201456 |
| Alexa Fluor Anti-Mouse 488 | Invitrogen | A11029 |
| Alexa Fluor Anti-Rabbit 488 | Invitrogen | A11034 |
| Alexa Fluor Anti-Rabbit 568 | Invitrogen | A11036 |
| Alexa Fluor Anti-Mouse 568 | Invitrogen | A11031 |
| Nucleophosmin | Abcam | ab37659 |
| Musashi1 | SantaCruz (IP) | SC-135721 |
| **Chemicals, Peptides, and Recombinant Proteins** | | |
| Importazole | Abcam | Ab146155-5mg |
| DMEM | Gibco | 11965-092 |
| Antibiotics | Gibco | 15240-062 |
| FBS | Gibco | 16000-044 |
| Paraformaldehyde, 4% | Electron Microscopy Sciences | 15714-S |
| Triton-X 100 | Sigma | T8787 |
| Protease Inhibitor | Thermo Scientific | A32953 |
| Tween | Fisher Scientific | BP337-500 |
| FBS-depleted DMEM | Gibco | LS11965118 |
| PBS | Corning | 46-013-CM |
| Gibco^TM^ Trypsin-EDTA, 0.25% Phenol red | Fisher Scientific | LS25200114 |
| Goat serum | Sigma | G90223-10mL |
| Accutase | Sigma-Aldrich | A6964-100ML |
| B27 Supplemental | Gibco | A3582801 |
| Amphotericin B | Gibco | 15290018 |
| NeurobasalTM medium | Gibco | 12348017 |
| L-glutamine | Hyclone | SH30034.01 |
| **Critical Commercial Assays** | | |
| Qproteome Cell Compartment Kit | Qiagen | #37502 |
| Alexa Fluor™ 568 NHS Ester | Invitrogen | A20003 |
| Lipofectamine^TM^ RNAiMAX Transfection Reagent | Thermo Scientific | 13778030 |
| PierceTM Co-Immunoprecipitation Kit | Thermo Scientific | #26149 |
| Micro BCA Kit | Thermo Scientific | #23235 |
| MSI1 Gapmers (5’ FAM labeled) | QIAGEN LLC | LG00214872-EDB |
| **Experimental Models: Cell Lines** | | |
| HEK-293 | Dr. Laura Blair |  |
| WT-6 tau iHEK | Dr. Laura Blair |  |
| P301L tau iHEK | Dr. Laura Blair |  |
| **Software and Algorithms** | | |
| GraphPad Prism 6 | Graphpad.com |  |
| ImageJ FIJI | ImageJ | NIH |
| Analyzer BZ-X | Keyence Microscope | Keyence Company |
| Arivis Vision 4D – 3D Viewer | Arivis AG | www.arivis.com |
| **Other** | | |
| 10kDa Amicon Ultra-0.5 Centrifugal Filter Units | Millipore | UFC501096 |
| Prolong Gold Antifade mounting media with DAPI | Invitrogen | P36941 |
| **Animal** |  |  |
| Tg(Prnp-MAPT*P301L)JNPL3Hlmc) | Taconic Biosciences | Model 1638 |
